# DNA dynamics in complex coacervate droplets and micelles†

**DOI:** 10.1039/d1sm01787j

**Published:** 2022-02-17

**Authors:** Inge Bos, Eline Brink, Lucile Michels, Joris Sprakel

**Affiliations:** Physical Chemistry and Soft Matter, Wageningen University & Research Stippeneng 4 6708 WE Wageningen The Netherlands joris.sprakel@wur.nl

## Abstract

Single stranded DNA (ssDNA), or another polyanion, can be mixed with polycations to form liquid-like complex coacervates. When the polycations are replaced by cationic–neutral diblock copolymers, complex coacervate core micelles (C3Ms) can be formed instead. In both complex coacervates and C3Ms, dynamics plays an important role. Yet, to date, the effect of chain length on the dynamics effect is still not fully understood. The DNA complexes provide a versatile platform to further elucidate these chain length effects because the DNA is monodisperse and its length can be easily adapted. Therefore, we study in this paper the dynamics of fluorescently labelled ssDNA in both complex coacervate droplets and micelles. The DNA dynamics in the complex coacervate droplets is probed by fluorescence recovery after photobleaching (FRAP). We observe that the DNA diffusion coefficient depends more strongly on the DNA length than predicted by the sticky Rouse model and we show that this can be partly explained by changes in complex coacervate density, but that also other factors might play a role. We measure the molecular exchange of C3Ms by making use of Förster resonance energy transfer (FRET) and complement these measurements with Langevin dynamics simulations. We conclude that chain length polydispersity is the main cause of a broad distribution of exchange rates. We hypothesise that the different exchange rates that we observe for the monodisperse DNA are mainly caused by differences in dye interactions and show that the dye can indeed have a large effect on the C3M exchange. In addition, we show that a new description of the C3M molecular exchange is required that accounts among others for the effect of the length of the oppositely charged core species. Together our findings can help to better understand the dynamics in both specific DNA systems and in complex coacervate droplets and micelles in general.

## Abstract 2

DNA can be mixed with oppositely charged homopolymers or diblock copolymers to form respectively complex coacervate droplets or complex coacervate core micelles. We study the chain length effect on the dynamics of these complex coacervate structures.

## Graphical abstract

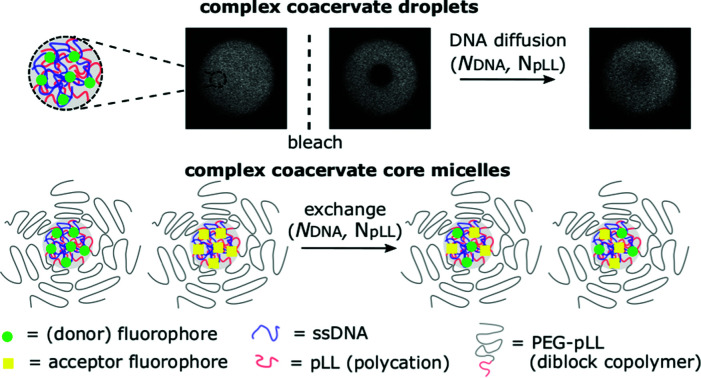

## 1 Introduction

DNA is a negatively charged polyelectrolyte and can form electrostatic complexes with positively charged macro-ions. This principle underlies for example DNA condensation in chromatin where the DNA binds to positively charged histone proteins.^1,2^ In addition, the formation of membraneless organelles inside cells is often based on liquid–liquid phase separation of nucleic acids with oppositely charged proteins.^3–5^ This liquid–liquid phase separation also occurs for synthetic oppositely charged polyelectrolytes and the liquid phase formed by the polyelectrolyte complex is usually called a complex coacervate. Apart from their ability to phase separate, also other properties of the membraneless organelles have recently been mimicked in artificial systems using both natural and synthetic polyelectrolytes,^5^ for example their ability to enhance catalysis^6,7^ or their ability to form multiple phases in one complex coacervate droplet.^8,9^

When DNA is mixed with a cationic–neutral diblock copolymer, it can form complex coacervate core micelles (C3Ms) instead of complex coacervate droplets: the repulsive interactions between the neutral blocks prevent further growth of the complex coacervate and in this way thermodynamically stable nanostructures can be formed instead of the macroscopic complex coacervates that will eventually form when DNA is mixed with polycations without neutral blocks. The C3M core is formed by the DNA and the cationic blocks, while the neutral blocks form the micelle corona. The micelle corona forms a protective layer around the micelle core and prevents micelle coalescence.^10^ In this way, micelles with specific polymer aggregation numbers and sizes of typically 10 to 100 nm are formed^11–13^ that can protect their core components from external components. Their protective properties as well as their well-defined small size makes these DNA complex coacervate core micelles promising DNA-based medicine delivery tools.^14–17^

Studying the liquid-like DNA polyelectrolyte complexes is not only useful to better understand the formation of membraneless organelles and to improve the formation of DNA-based medicine delivery tools, but also to give fundamental insights into polyelectrolyte complexes in general. In particular, the effect of chain length and chain length polydispersity can be systemically studied with DNA because specific DNA sequences can be synthesised. This yields monodisperse DNA whose length can be systematically varied by changing the sequence. At the moment, the chain length effects on the complex coacervates and C3Ms are not completely understood. Especially the chain length effect on the coacervate dynamics is not well understood, as we will explain below.

The dynamics of complex coacervates determines the response of complex coacervate materials to deformation^18,19^ and might determine their response time to dissociation triggers.^20^ The chain length effect on the dynamics in complex coacervates is usually described by the sticky Rouse model^21^ where the ionic bonds act as sticky points that slow down the dynamics.^18,19^ In the sticky Rouse model, the overall polymer relaxation time scales with the squared number of intermolecular sticky bonds, which means for polyelectrolytes that the relaxation time should scale with the square of the polyelectrolyte length. For complex coacervates with matched chain length this description seems to work well,^18^ but for complexes where the lengths of the anionic and cationic chain differ, the situation is more complex. In some cases, the dynamics seems to be solely governed by one of the two polyelectrolytes, while the length of the other polyelectrolyte does not have any effect.^18^ Since only few studies have focused on polyion pairs with incommensurate lengths, an understanding of these asymmetry effects, and with that a complete description of coacervate dynamics, is lacking.

The molecular exchange dynamics of complex coacervate core micelles determines how often the core components are exposed to the surroundings and in this way the level of protection that the C3M offers to a cargo that it encapsulates. We have previously used Förster resonance energy transfer (FRET) to measure this molecular exchange of C3Ms and observed a broad range of exchange rates,^22^ similar to what has been observed earlier for other C3Ms.^23^ We hypothesised that this large difference in exchange rates is the result of chain polydispersity. Recently, the molecular exchange of C3Ms was also measured by small angle neutron scattering (SANS) and also here the presence of different exchange rates was explained by chain polydispersity.^24^ However, this polydispersity hypothesis has not been confirmed yet, since so far only the exchange of polydisperse polymers has been measured. Measuring the exchange of C3Ms with ssDNA allows to thoroughly test this hypothesis because the DNA is monodisperse and its length can be systematically varied.

**Fig. 1:**
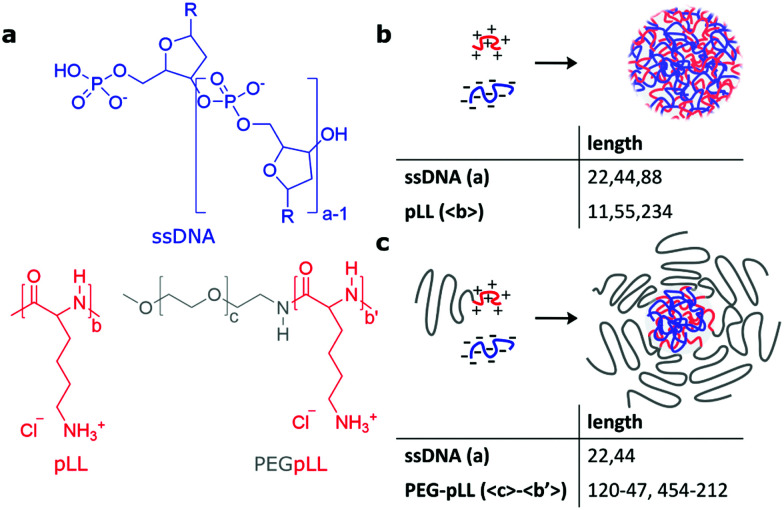
Schematic overview of the complex coacervate droplet and micelle formation. (a) The chemical structures of ssDNA, poly(l-lysine) (pLL) and poly(ethyleneglycol)–poly(l-lysine) (PEG–pLL). The R in the chemical structure of ssDNA indicates a nucleobase (adenine, guanine, cytosine or thymine). The subscripts a, b, b′ and c indicate the degree of polymerization of ssDNA, pLL, the pLL block and the PEG block respectively. (b) Schematic overview of the formation of complex coacervate droplets and the polymer lengths that are used to form these droplets. (c) Schematic overview of the formation of complex coacervate core micelles and the polymer lengths that are used to form these micelles.

Apart from the limited knowledge on the chain lengths effect on the dynamics in both complex coacervates and C3Ms, also little is known on how the dynamics inside bulk coacervates compares to that in the nanoscopically confined interior of C3Ms, and to what extent the dynamics in bulk coacervates is predictive for the exchange dynamics in micelles. Therefore, we study in this paper the dynamics of single stranded DNA in both complex coacervate droplets and micelles (Fig. 1) and we also compare these systems. For the complex coacervates droplets, we use fluorescence recovery after photobleaching (FRAP) to measure the diffusion coefficient of fluorescently labelled ssDNA. For the C3Ms, we use FRET-based exchange measurements to follow the exchange of both the ssDNA and the diblock copolymers and we complement these measurements with Langevin dynamics simulations. In this way, we show that both the DNA diffusion in complex coacervates and the DNA exchange of C3Ms depend on both the DNA chain length and the cationic chain length. We observe that the DNA diffusion coefficient shows a stronger dependence on the DNA length than predicted by the sticky Rouse model, which can only be partially explained by the chain length effect on coacervate density. In addition, we conclude from the comparison of the exchange of the monodisperse ssDNA and the polydisperse diblock copolymer that chain length polydispersity is indeed the main factor underlying the earlier observed broad distribution of exchange times. We hypothesise that the different exchange rates that we observe for the monodisperse DNA are mainly caused by differences in interactions of the donor and acceptor fluorophore label and show that the fluorophore can indeed have a large effect on the C3M exchange. Finally, we discuss a new description of the C3M exchange to account among others for the effect of the chain length of the oppositely charged core species on the exchange rate. Together our results can help to better understand both DNA specific systems and complex coacervates droplets and micelles in general.

## 2 Materials and methods

### 2.1 Materials

The unlabelled single stranded DNA oligonucleotides and the fluorescently labelled oligonucleotides with a donor (cyanine3 or atto488) or acceptor (cyanine5 or atto532) dye attached to the 5′ end were purchased from Integrated DNA Technologies. The oligonucleotide sequences (Table S1, ESI†) were the same as described by Lueckheide *et al.*^13^ These sequences are based on a sequence complementary to human microRNA-21 and are designed in such a way that the secondary structure formation and self-dimerisation is minimised. Stock solutions of oligonucleotides were prepared by resuspending in nuclease free water at a monomer concentration of 10 mM based on the absorption measurements by the manufacturer.

The poly(l-lysine) homopolymers with number averaged lengths of 11, 55 and 234 amino acids (pLL11, pLL55 and pLL234 respectively) and the poly(ethyleneglycol)(5k)-*b*-poly(l-lysine)_47_ (PEG-5k-pLL47, where 5k indicates the 5300 g mol^−1^ molecular weight of the poly(ethyleneglycol) block and 47 indicates to the number averaged amino acid length of the poly(l-lysine) block) and poly(ethyleneglycol)(20k)-*b*-poly(l-lysine)_212_ (PEG-20k-pLL212) diblock copolymers were purchased from Alamanda Polymers as chloride salts. The overall polydispersities of the diblock copolymers as measured by the manufacturer were 1.06 for PEG-5k-pLL47 and 1.03 for PEG-20k-pLL212. Stock solutions of pLL homopolymers and diblock copolymers were prepared by dissolving in 1× phosphate-buffered saline (137 mM NaCl, 2.7 mM KCl, 8 mM Na_2_HPO_4_, 2 mM KH_2_PO_4_, pH 7.4) (PBS) and subsequent sonication for 10 minutes, as recommended by the manufacturer.

Sulfo-cyanine 3 NHS ester (sCy3) and sulfo-cyanine 5 NHS ester (sCy5) dyes for PEG-5k-pLL47 labelling were purchased from Lumiprobe.

### 2.2 PEG–pLL labelling

Fluorescently labelled PEG-5k-pLL47 was obtained by using a NHS coupling reaction between the amino groups of the poly(l-lysine) block and the sCy3 or sCy5 dye. The PEG-5k-pLL47 and the sulfo-cyanine dye (either sCy3 or sCy5) were dissolved in 1× PBS to give a final lysine monomer concentration of 25 mM and a dye concentration of 0.5 mM. This corresponds to a maximum label degree of on average 1 dye per polymer chain, but the coupling efficiency is not 100% and therefore the average numbers of dyes per polymer chain is lower than 1. The reaction mixture was stirred for 4 hours at room temperature. Subsequently, the reaction mixture was dialysed for several days and afterwards the sample was freeze-dried to obtain the fluorescently labelled PEG-5k-pLL47-sCy3 and PEG-5k-pLL47-sCy5.

### 2.3 FRAP measurements

Complex coacervate samples for fluorescence recovery after photo-bleaching (FRAP) measurements were prepared by mixing stock solutions of oligonucleotides labelled with cyanine3, unlabelled oligonucleotides of the same length and poly(l-lysine) in 1× PBS. To minimise the effect of dye interactions, the fraction of labelled oligonucleotides *α* was only 0.002, 0.004 and 0.008 for the nt22, nt44 and nt88 nucleotides respectively. For the nt44 and nt88 nucleotides, an anionic and cationic monomer concentration of 2.5 mM was used (which corresponds to a charge ratio of 1 : 1). For the nt22 nucleotides, both the anionic and cationic monomer concentration was increased to 5 mM to ensure that also for these samples large enough droplets were formed to analyse the FRAP data with an infinite medium diffusion model (*i.e.* the droplet size is larger than 2.3 times the bleached spot size).^25^ The samples were prepared directly at microscopy glass slides coated with a 5 wt% poly(vinyl alcohol) solution, sealed by gluing a cover slip on top of the slide and measured 1 to 4 h after preparation. The FRAP measurements were performed on a Nikon C2 laser scanning confocal microscope with a 60× 1.4 NA oil-immersion objective. A 488 nm laser was used for both imaging and bleaching. The images were recorded at a 4× digital zoom and consisted of 512 × 512 pixels. Bleaching of a spherical region with a 3 μm diameter was performed in the middle of a coacervate droplet. The 3D spherical shape of the bleach profile, which is required for the data analysis with the 3D diffusion model,^25^ was confirmed by a z-stack measurement acquired immediately after bleaching a 3 μm diameter region of a coacervate droplet of a nt88/pLL234 sample (Fig. S1a and b, ESI†). Ten images were recorded before bleaching and 301 images were recorded after bleaching to follow the recovery. For the nt44 and nt88 samples, the imaging interval was 2 s and for the nt22 samples the imaging interval was 1.4 s.

Image analysis of the FRAP data was based on the method described by Taylor *et al.*^25^ The fluorescence intensity concentration *C* inside the bleach spot was normalised according to:1
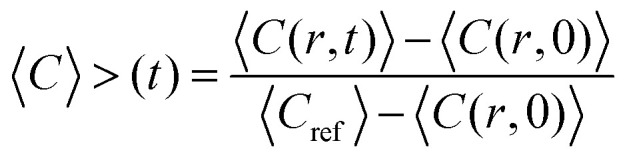
where 〈*C*(*r*,*t*)〉 is the average fluorescence intensity in the bleach spot at a time *t* after bleaching and *C*_ref_ is the average reference intensity in the bleach spot before bleaching. To account for movements of the coacervate droplets, the position of the center of the bleach spot was determined for every image in the recovery sequence separately. During the recovery, some additional bleaching occurred. Corrections for this bleaching were calculated from the intensity of a coacervate region in the image outside the bleached spot.

To obtain the characteristic diffusion time *τ*_D_, the bleaching corrected data was fitted with a 3D diffusion model in an infinite medium:^25^2
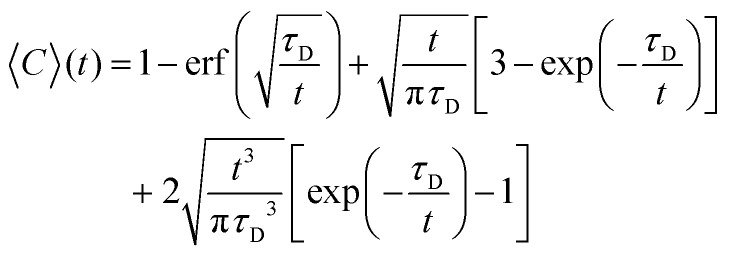
The diffusion coefficient *D*^app^ was calculated from the obtained characteristic time *τ*_D_ by *D*^app^ = *R*^2^/*τ*_D_ where *R* is the radius of the bleach region. The reported diffusion coefficients are the average of the diffusion coefficient of FRAP measurements at five different positions in the sample. For all fits, we observed a small systematic deviation between the fit and the data at the start of the recovery curve with the fit showing a slightly faster recovery than the data. This is probably because the bleaching by the laser was not perfect and also some molecules outside the bleach spot were bleached, as shown by a slight decrease in intensity outside the bleach spot directly after bleaching compared to the same position directly before bleaching (Fig. S1c, ESI†). In the article of Taylor *et al.*,^25^ they have tried to correct for this by introducing a shift factor in the fit of the bleach profile that accounts both for imperfect bleaching by a laser with Gaussian profile and for recovery during the bleaching. However, in this case the bleach profile is not Gaussian and cannot be fitted by simply introducing a shift factor (Fig. S1c, ESI†) and therefore we have not used this correction method. Because of the imperfect bleaching, the values of the reported apparent diffusion coefficients might slightly deviate from the real diffusion coefficients, but since we have used the same bleaching method for the different DNA and pLL lengths, we do not expect large deviations in the ratios of the diffusion coefficient.

### 2.4 Sample preparation for complex coacervate microviscosity measurements

The microviscosity inside the complex coacervate droplets was probed by measuring the fluorescence lifetime of a sulfonated boron-dipyrromethene (sulfo-BODIPY) molecular rotor in these droplets. The synthesis of this sulfo-BODIPY molecular rotor is described elsewhere.^26^ Complex coacervate droplets containing sulfo-BODIPY molecular rotors were prepared by mixing water, concentrated PBS, oligonucleotides, poly(l-lysine) and sulfo-BODIPY stock solution (1 g L^−1^ in water) to reach in a 1× PBS solution a cationic and anionic monomer concentration of 2.5 mM (corresponding to a charge ratio of 1 : 1) and a sulfo-BODIPY concentration of 10 μM. The samples were prepared directly at microscopy glass slides coated with a 5 wt% poly(vinyl alcohol) solution, sealed by gluing a cover slip on top of the slide and measured 1 to 3 h after preparation (Section 2.6).

### 2.5 Sample preparation for FRET-FLIM measurements

Complex coacervate droplets with an atto488 and atto532 FRET pair were prepared by first mixing unlabelled and fluorescently labelled nt44 oligonucleotides to get a fraction of labelled chains *α* of 0.06. Half of the fluorescent labels was atto488 and the other half of fluorescent labels was atto532. Subsequently, concentrated PBS solution and poly(l-lysine) were added to get a final monomer cationic and anionic monomer concentration of 2.5 mM (corresponding to a charge ratio of 1 : 1) in a 1× PBS solution. To determine the fluorescence lifetime of the atto488 donor in absence of the atto532 acceptor, a similar sample preparation protocol was used, but in this case the label fraction *α* was 0.03 and all fluorescent labels were atto488. In all cases, the samples were prepared directly at microscopy glass slides coated with a 5 wt% poly(vinyl alcohol) solution, sealed by gluing a cover slip on top of the slide and measured 1 to 3 h after preparation (Section 2.6). All FRET-FLIM samples were prepared and measured *in duplo*.

### 2.6 FLIM measurements

Fluorescence lifetime image microscopy (FLIM) measurements of both the fluorescence of sulfo-BODIPY molecular rotors in the complex coacervate samples (Section 2.4) and the fluorescence of atto488 donors in complex coacervate samples (Section 2.5) were performed on Leica TCS SP8 inverted scanning confocal microscope coupled with a Becker–Hickl SPC830 time-correlated single photon counting (TCSPC) module. A 63× 1.2 NA water immersion object was used for imaging. Images of 256 × 256 pixels were recorded at a line scanning speed of 400 Hz. The acquisition time was set to 80 s for each image. The samples were excited at 488 nm by a pulsed white light laser with a frequency of 40 MHz. The emission was collected using a spectral window of 20 nm centered on 510 nm (atto488) or 525 nm (sulfo-BODIPY) onto a Leica HyD SMD hybrid photodetector. For each sample, a FLIM image was recorded at three different positions in the sample.

After image acquisition SPCImage 8.4 software (Becker & Hickl) was used to fit the fluorescence decay curves in each pixel. A two-component exponential decay was used to determine the average fluorescence lifetime in each pixel. A false colour scale was used to report lifetime values and yield the fluorescence lifetime images.

The majority of the sulfo-BODIPY partitioned inside the complex coacervate. Yet, a small fraction of the sulfo-BODIPY remained free in solution. To exclude the fluorescence lifetime of these free sulfo-BODIPY molecular rotors from the lifetime comparison, the lifetimes of pixels with a fluorescence intensity 2.5 times smaller than the average image intensity were excluded from the lifetime histograms. No atto488 fluorescence was detected outside the complex coacervate droplets and therefore the fluorescence lifetime histograms of the atto488 dye could be directly obtained from the FLIM images.

### 2.7 Micelle preparation

Micelles were prepared based on the thermal annealing protocol of Lueckheide *et al.*^13^ For the micelles with fluorescently labelled DNA, stock solutions of fluorescently labelled oligonucleotides and unlabelled oligonucleotides of the same length were mixed with nuclease free water and concentrated PBS solution to get the desired fraction of labelled chains *α* and to give the desired final PBS concentration. Subsequently, the PEG–pLL diblock copolymer stock solution was added to give a charge unit concentration of 1 mM for both the oligonucleotides and the PEG–pLL diblock copolymers (the charge ratio is 1 : 1). The samples were immediately vortexed for 20 s and subsequently heated at 75 °C for 2 h. Samples were cooled to room temperature and measured after being at least 1 h at room temperature. Unless otherwise indicated, a label fraction of *α* = 0.03 was used for the nt22/PEG–pLL micelles and *α* = 0.06 was used for the nt44/PEG–pLL micelles. We note that at this label fraction, already some self-quenching occurs for the micelles labelled with atto488 or atto532 (Fig. S4, ESI†). However, a lower label fraction resulted in a low FRET efficiency where the FRET was difficult to distinguish from the direct acceptor excitation, especially for the micelles in 1× PBS, and therefore this lower label fraction was not used.

For the micelles with fluorescently labelled PEG-5k-pLL47 the same preparation protocol was used. The only difference is that labelled and unlabelled PEG-5k-pLL47 diblock copolymers were first mixed in the desired ratio and that afterwards the oligonucleotides were added. In this case, 10% of the PEG-5k-pLL47 polymers was coming from the fluorescently labelled PEG-5k-pLL47 stock solution and the other 90% was coming from the unlabelled PEG-5k-pLL47 stock solution with the same monomer concentration. The exact label fraction corresponding to this mix ratio is not known because we do not know the coupling efficiency of the PEG–pLL labelling reaction, but it is lower than 0.10 since the maximum average amount of fluorescent labels per polymer chain is 1 for this reaction.

The ssDNA/PEG–pLL micelles in 0.2× PBS solution were also prepared following the same thermal annealing protocol. For these micelles, only unlabelled ssDNA and PEG–pLL were added and no fluorescently labelled polymers were used.

### 2.8 Light scattering measurements

Static light scattering measurements of the ssDNA/PEG–pLL micelles were performed on an ALV instrument equipped with a 660 nm laser over a detection angle range from to 46° to 146° in intervals of 2°. Five runs of 30 s were performed at every detection angle. The Rayleigh ratio *R* at each detection angle *θ* was calculated by:3
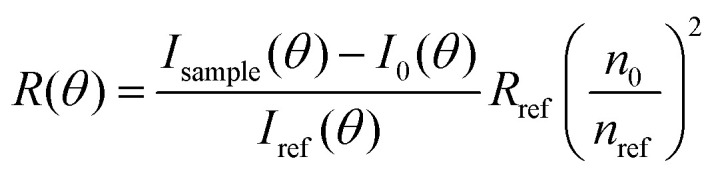
Here *I*_sample_(*θ*), *I*_0_(*θ*) and *I*_ref_(*θ*) are the sample, solvent and reference scattering intensities respectively, *n*_0_ and *n*_ref_ are the refractive index of the solvent and reference respectively and *R*_ref_ is the Rayleigh ratio of the reference. The refractive index of the solvent is *n*_0_ = 1.3332. We have used toluene as reference with *n*_ref_ = 1.496 and *R*_ref_ = 8.56 × 10^−4^ m^−1^.^27^ The micelle molar mass was estimated based on both Zimm analysis and Guinier analysis of the measured Rayleigh ratio at different scattering angles. According to the Zimm approximation, the Rayleigh ratio is given by:4
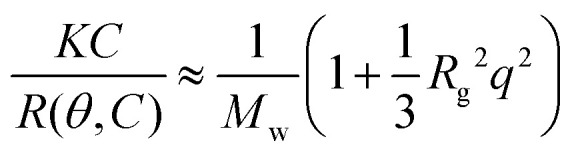
where *C* is the mass concentration, *M*_w_ is the molar mass of the scattering particle, *R*_g_ is its radius of gyration, *q* = (4π*n*_0_/*λ*)sin(*θ*/2) is the scattering vector with *λ* the laser wavelength and *K* is an optical constant given by:5
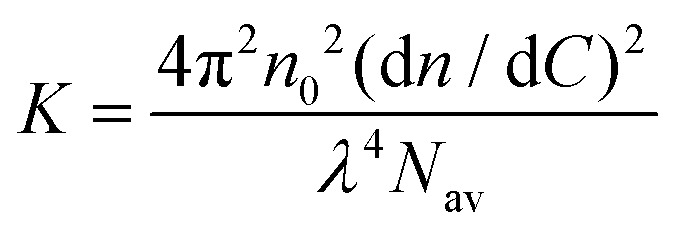
where *N*_av_ is Avogadro's number and d*n*/d*C* is the specific refractive index increment of the micelles, for which we have used a weighted average of the refractive index increments of PEG, poly(l-lysine) and DNA, which are 0.135 mL g^−1^, 0.17 mL g^−1^ and 0.17 mL g^−1^ respectively.^28,29^ In the Guinier approximation, the Rayleigh ratio is given by:6
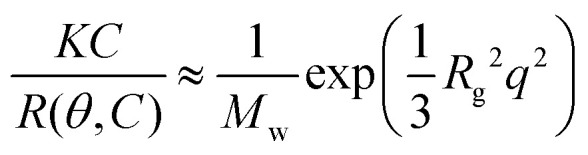
Both the Zimm and Guinier approximation can thus be used to obtain the molar mass of the micelle and therefore the micelle aggregation number. Data points measured at scattering angles below 70° and above 120° deviated from the linear dependence in both the Zimm and Guinier plot (Fig. S3, ESI†) and were therefore excluded from the data analysis, like we also did previously.^22^

### 2.9 Fluorescence spectroscopy measurements

Fluorescence spectroscopy measurements were performed on an Agilent Cary Eclipse fluorescence spectrophotometer connected to a PCB-150 circulating water bath. All measurements were performed at 20 °C. We note that the temperature control is essential because the dye quantum yield can depend strongly on the temperature^30^ and in that case small temperature fluctuations can cause significant fluctuations in FRET efficiency.

An excitation wavelength of 490 nm was used to excite the (sulfo-)cyanine 3 donor dye. At this wavelength, the direct excitation of the (sulfo-)cyanine 5 acceptor dye is negligible. An excitation wavelength of 470 nm was used to excite the atto488 dye. To calculate the FRET efficiency, the emission of the atto532 acceptor dye was corrected for direct acceptor excitation at this wavelength. The degree of self-quenching of the atto532 acceptor dyes in the micelles was determined by using an excitation wavelength of 510 nm.

For the measurements of the equilibrated micelles, a single emission spectrum of the equilibrated sample was recorded. For the micelle exchange measurements, the equilibrated donor and acceptor micelles solutions were added to the cuvette in a 1 to 1 concentration ratio and mixed and placed in the spectrophotometer. Every minute an emission spectrum was recorded.

To determine the FRET efficiency, the spectrum (after correction for direct acceptor excitation for the atto488/atto532 FRET pair) was fitted with a linear combination of fixed log normal functions to determine the relative contribution of the donor and acceptor emission to the overall emission spectrum (ESI,† Section S3). Finally, the FRET efficiency *E* was calculated by:7
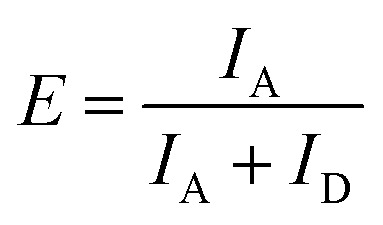
where the donor intensity *I*_D_ and acceptor intensity *I*_A_ follow from integration of the donor and acceptor part of the emission spectrum respectively.

### 2.10 Fit of the FRET-based C3M exchange experiments to analytical model

To explain the shape of the FRET-based exchange experiment curve for ssDNA/PEG–pLL micelles with atto488/atto532 labels in 0.2× PBS solution, we have fitted the exchange experiment data with the analytical FRET model that we have developed recently.^22^ Briefly, we have first determined the micelle geometric constant *ν* by fitting the FRET efficiency at different fractions of labelled chains (Fig. S5, ESI†) to the function:8
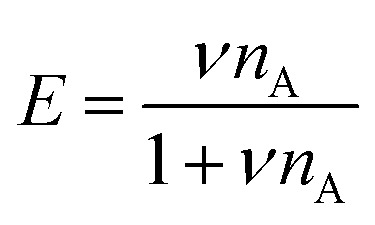
where *n*_A_ is the average number of acceptor fluorophores per micelle, which can be calculated by *n*_A_ = 0.5*Nα* where *N* is the total number of ssDNA chains in the micelle, which is measured by static light scattering experiments (Section 2.8), and *α* is the fraction of labelled chains (half of the labelled chains were acceptors and therefore *α* is multiplied by 0.5).

Subsequently, the data of the C3M exchange experiments is fitted to the following function:9
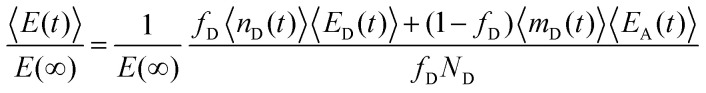
Here, *E*(∞) is the FRET efficiency of the completely mixed micelles and is for the right hand side of the equation given by eqn (8) with *n*_A_(∞) = (1 − *f*_D_)*Nα* where *f*_D_ is the fraction of micelles containing only donors at the start of the mixing experiments (*f*_D_ = 0.5 in all mix experiments in this paper) and *α* is the label fraction used in the mix experiment. We have divided by *E*(∞) at both sides of eqn (9) because experimental variations can result in a slightly different final FRET efficiency compared to the theoretical FRET efficiency. If the experimental FRET efficiency is slightly lower than the theoretical FRET efficiency of the completely mixed micelles, this will introduce an artificially low exchange time in the fit when directly comparing the FRET efficiency *E*(*t*) and therefore we compare the normalised FRET efficiencies *E*(*t*)/*E*(∞) instead. 〈*n*_D_〉 is the average number of donors in micelles that contained only donors at the start of the mixing experiment (the donor micelles) and 〈*m*_D_〉 is the average number of donors in micelles that contained only acceptors at the start of the mixing experiments (the acceptor micelles). The average number of donors in donor micelles at a certain time *t* is given by:10
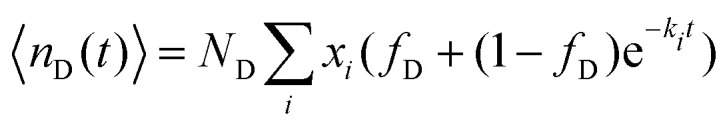
where *N*_D_ is the average number of donors in donor micelles at the start of the mixing experiment, *k*_*i*_ is the exchange rate of donor species *i* and *x*_*i*_ indicates the fraction of this species compared to all donor species, such that 
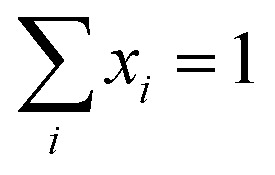
.

The average number of donors in acceptor micelles at a certain time *t* is given by:11
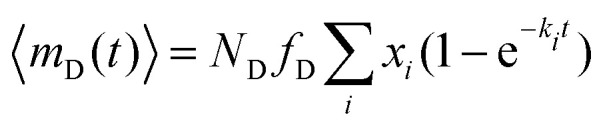
〈*E*_D_(*t*)〉 and 〈*E*_A_(*t*)〉 in eqn (9) are the average FRET efficiency at a time *t* in the donor micelles and acceptor micelles respectively. They are given by:12
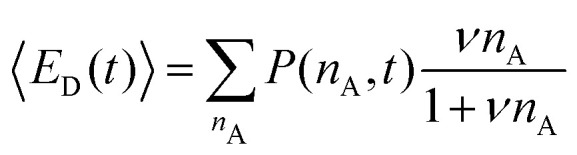
and13
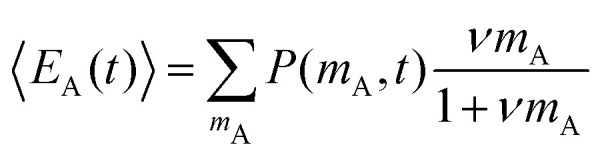
where *P*(*n*_A_,*t*) is the probability to have *n*_A_ acceptors in donor micelles at time *t* and *P*(*m*_A_,*t*) is the probability to have *m*_A_ acceptors in acceptor micelles at time *t*. *P*(*n*_A_,*t*) is given by:14
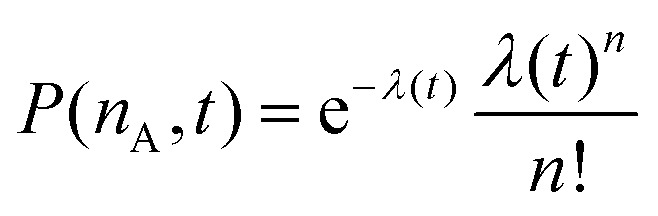
where *n* is the shortened notation of *n*_A_ and *λ*(*t*) is given by:15
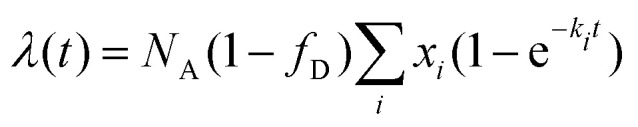
where *N*_A_ is the average number of acceptors in acceptor micelles at the start of the mixing experiment, *k*_*i*_ is the exchange rate of acceptor species *i* and *x*_*i*_ indicates the fraction of this species compared to all acceptor species, such that 
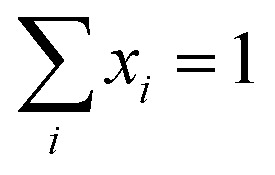
. *P*(*m*_A_,*t*) is given by:16
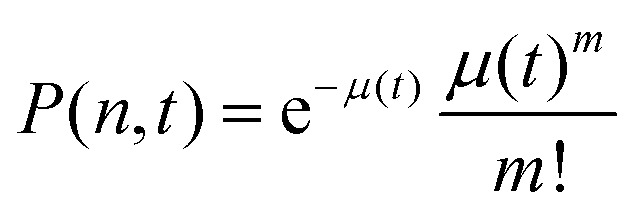
where *m* is the shortened notation of *m*_A_ and *μ*(*t*) is given by:17
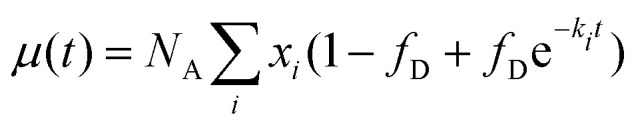


In this case, we have used two different exchange rates for both the donor and acceptor exchange. We imposed that the fraction of fast exchanging fluorophores *x*_fast_ was the same for the donors and acceptors. In addition, we required that the fast exchange rate *k*_fast_ was the same for the donor and the acceptor, while the slow exchange rate of the donor and acceptor could be different. This resulted in four different fit parameters: *x*_fast_, *k*_fast_, *k*_D_ and *k*_A_, where *k*_D_ and *k*_A_ indicate the slow exchange rate of the donor and acceptor respectively. The fits were performed by using a nonlinear least-square fit algorithm, as implemented in the python software package SciPy, with the following boundaries for the fit parameters: *x*_fast_ = [0,0.2], *k*_fast_ = [10 h^−1^, 1000 h^−1^], *k*_D_ = [0 h^−1^, 100 h^−1^] and *k*_A_ = [0 h^−1^, 100 h^−1^]. For the initial guess of *k*_D_ we have used a value twice as large as the initial guess of *k*_A_ (in all cases, *k*_D_ = 0.02 h^−1^ and *k*_A_ = 0.01 h^−1^) since the use of *k*_D_ = *k*_A_ as initial guess decreased the quality of the fit with *E*(*t*) = *E*(∞) being reached too early.

### 2.11 Langevin dynamics simulations

Langevin dynamics simulations were based on the same approach as described earlier:^31^ the Kremer–Grest bead-spring model in combination with Coulomb interactions was used to simulate coarse-grained cationic–neutral diblock copolymers and negatively charged homopolymers. Counter ions were modelled as beads with the same size of the monomers. No additional salt ions were added. All beads have a size *σ*_s_ and a mass *m*_s_. The Bjerrum length *l*_B_ in the Coulomb potential was set to *l*_B_ = 2.5*σ*_s_. The polymer bonds are modelled with a finitely extensible nonlinear elastic (FENE) potential with a bond stiffness *k*_bond_ of 30*k*_B_*T*/*σ*_s_^2^ and a maximum bond extension distance *r*_bond_ of 1.5*σ*_s_. The non-electrostatic interactions between equally and oppositely charged monomers was modelled with a Lennard-Jones potential with a cutoff distance of 2.5*σ*_s_ and a Lennard-Jones minimum *ε*_LJ_ of 0.05*k*_B_*T*. The non-electrostatic interaction between all other monomer–monomer, monomer–ion, and ion–ion combinations was purely repulsive. For this repulsive interaction we used the Weeks–Chandler–Andersen (WCA) potential with an interaction strength *ε* = 1*k*_B_*T*.

For all 223 diblock copolymers in the simulation a neutral block length of 50 monomers was used. The cationic block was polydisperse and 48 diblock copolymers had a cationic block length of *N*_pos_ = 14, for 47 diblock copolymers *N*_pos_ = 16, for 40 diblock copolymers *N*_pos_ = 18, for 40 diblock copolymers *N*_pos_ = 20 and for 48 diblock copolymers *N*_pos_ = 22. The polyanion was monodisperse. Two different simulations were performed: one with 400 polyanions with a length of *N*_neg_ = 10 and one with 200 polyanions with *N*_neg_ = 20. In both cases, the periodic box size was *L*_s_ = 163*σ*_s_.

We started the simulations by placing the homopolymers, diblocks, and counterions randomly in the simulation box and used the GPU-optimized molecular dynamics software package HOOMD-blue^32–35^ to perform the Langevin dynamic simulations. The Coulomb interactions were calculated by using the particle–particle particle-mesh (PPPM) Ewald summation method^34^ with a real space cut off distance of 2.5*σ*_s_. The neighbour lists were generated by using the linear bounding volumes hierarchies (LBVHs) method.^35^ We used *γ* = 1*m*_s_/*τ*_s_ as drag coefficient and Δ*t* = 0.005*τ*_s_ as simulation time step where *τ*_s_ is the time unit in the system. We saved the configuration of the simulation every 2500 steps. The total simulation time was 2 × 10^7^*τ*_s_.

To determine the number of split events per polymer chain, the composition of the micelle clusters was followed over time. The micelle cluster identification was performed in the same way as described earlier.^31^ Every time a polymer was part of the smallest cluster formed after a splitting event of a micelle cluster, this was counted as a split event for this polymer. To reduce the effect of micelle growth on the split events analysis, the split events at a simulation time shorter than 2 × 10^6^*τ*_s_ were excluded from the analysis.

## 3 Results and discussion

### 3.1 DNA diffusion in complex coacervate droplets

The strength of the electrostatic bonds in complex coacervates is often on the order of the thermal energy and thermal fluctuations can thus continuously break and reform these electrostatic bonds. The transient nature of the electrostatic bonds makes the complex coacervate dynamic systems. In active coacervate droplets like membraneless organelles, this dynamic effect is even further increased by covalent changes of the phase separating molecules in time, but also in passive complex coacervate droplets dynamics already plays a large role. For example, the dynamics of a complex coacervate material determines its response to deformation. The complex coacervate dynamics is affected by the polyelectrolyte chain length. In general, increasing the chain length results in more ionic bonds per polyelectrolyte chain and therefore the relaxation of the complex coacervate becomes slower.^18,36,37^ Yet, more complex effects can occur when the oppositely charged polyelectrolytes have different lengths and their lengths are varied independently from each other: in some cases, only one of the two polyelectrolyte species determines the dynamic response.^18^ At the moment it is difficult to identify the origin of this asymmetric response because there are only a few studies that have quantitatively determined the chain length effects on the dynamics in complex coacervates with mismatched chain lengths.^18,37^

**Fig. 2:**
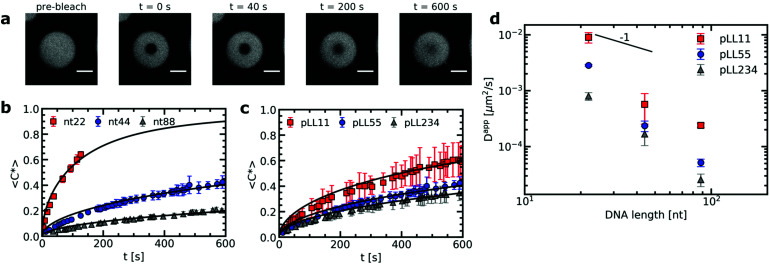
FRAP experiments of ssDNA/pLL complex coacervate droplets with fluorescently labelled ssDNA. (a) Example images at different time points of a FRAP experiment of a nt44/pLL55 complex coacervate droplet. Size scale bars correspond to 5 μm. (b) Comparison of the normalised fluorescence recovery curve for complex coacervates with pLL55 and different ssDNA lengths (nt22, nt44 and nt88) averaged over 5 different positions in the sample. For nt22, the data of the recovery curve stops after 150 s because from this point the contrast between the bleach spot and its surroundings becomes too low to track the center of the bleach spot. (c) Comparison of the average normalised fluorescence recovery curve for complex coacervates with nt44 and different pLL lengths (pLL11, pLL55 and pLL234) averaged over 5 different positions in the sample. Solid lines in (b) and (c) indicate the recovery curves calculated from the average diffusion coefficients obtained from fitting the individual recovery curves. (d) The average apparent diffusion coefficient *D*_app_, obtained from fitting the fluorescence recovery curves, as function of the ssDNA length.

Here we have used fluorescence recovery after photo-bleaching (FRAP) to systematically study the chain length effect on the diffusion of fluorescently labelled ssDNA in ssDNA/pLL coacervates (Fig. 2). We have varied both the pLL length and the ssDNA length independently from each other. The increase in FRAP recovery time for increasing chain lengths, shows that the DNA diffusion is not only affected by its own length (Fig. 2b), but also by the length of the oppositely charged poly(l-lysine) (Fig. 2c). This indicates that the relaxation time of a polyelectrolyte in a complex coacervate is not only determined by its own length and the ionic strength of the solution, as shown earlier,^18,19,38^ but that also other complex coacervate properties can affect the dynamics.

From the recovery curves the diffusion coefficient of the DNA in the complex coacervate droplets can be determined. Here, we have used the 3D diffusion model developed by Taylor *et al.*^25^ to obtain the apparent DNA diffusion coefficients, as explained in the materials and methods (Section 2.3). The DNA diffusion coefficient strongly decreases with increasing DNA length *N* (Fig. 2d). In fact, it decreases more strongly than the *D* ∝ *N*^−1^ dependence predicted by the sticky Rouse model, which is the model that is often used to describe polyelectrolyte dynamics in coacervates. Instead of an exponent of −1, we find an exponent of −2.6 ± 0.8, −2.9 ± 0.4 and −2.5 ± 0.1 for pLL11, pLL55 and pLL234 respectively. A possible explanation for this stronger decrease is that the density of the complex coacervate also increases with increasing DNA length and that this increase in density slows down the dynamics in the complex coacervate. This increase in density is predicted by multiple coacervation models^39,40^ and has been mentioned as explanation why the viscosity depends more strongly on the polyelectrolyte length than predicted by the sticky Rouse model.^18^ This density increase has also been mentioned to explain why the relaxation time in coarse-grained simulations of salt-free polyelectrolyte complexes of equal length showed a stronger dependence on the polyelectrolyte length than the sticky Rouse model.^41^ In the latter case, the increase in complex density was also confirmed by the same simulations. For the ssDNA/pLL complex coacervate droplets, an increase in density with increasing chain length might not only explain the stronger dependence of DNA diffusion coefficient on the DNA length, but it might also explain why the pLL chain length affects the DNA diffusion coefficient in the complex.

### 3.2 Chain length effect on the complex coacervate density

We have used a sulfonated boron-dipyrromethene (sulfo-BODIPY) molecular rotor^26^ to probe the changes in local coacervate density for different ssDNA and poly(l-lysine) chain lengths. This sulfo-BODIPY molecule is a rigidochromic sensor: its fluorescence lifetime depends on its rate of intramolecular rotation and this rate of intramolecular rotation depends on the mechanics of its surroundings. A higher intramolecular rotation rate makes the non-radiative fluorescence decay pathway more favourable and therefore the fluorescence lifetime of the molecular rotor is shorter. An increase the microviscosity can slow down the intramolecular rotation rate and thus can be observed by an increase in the fluorescence lifetime. Since the fluorescence lifetime is determined by rotations on the molecular scale, the molecular rotor will mainly probe changes in microviscosity caused by changes in the mesh size of the complex coacervate. This mesh size scales inversely with the polymer density in the complex coacervate: an increase in fluorescence lifetime corresponds to a decrease in mesh size and an increase in the density of the coacervate. The changes in microviscosity measured by the molecular rotor can be different from the macroscopic viscosity changes. Fist, the macroscopic viscosity is governed by the overall polymer relaxation rather than changes on the smaller mesh size scale. In addition, the macroscopic viscosity averages over all changes on the microscopic scale while with the molecular rotors inhomogeneities on the microscopic scale can still be distinguished.

**Fig. 3:**
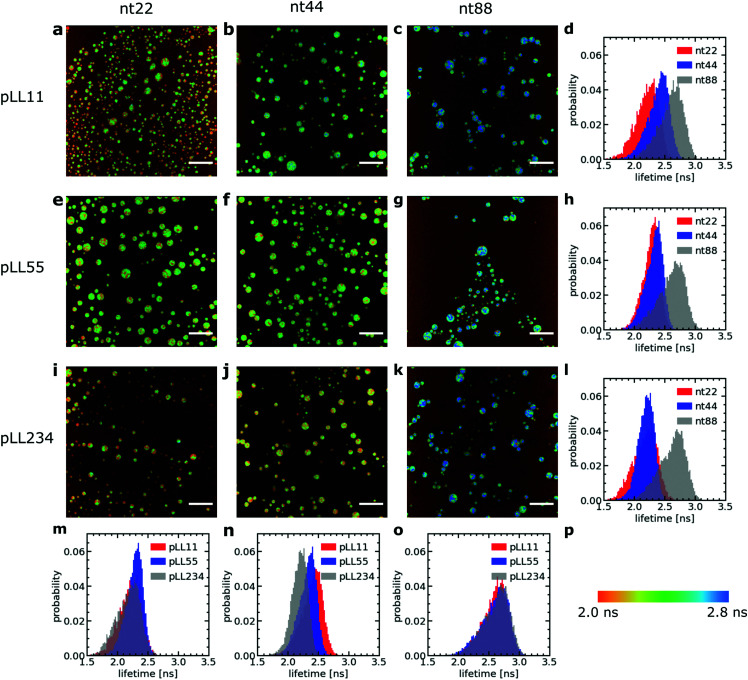
Fluorescence lifetime image microscopy (FLIM) measurements of a sulfo-BODIPY molecular rotor in ssDNA/pLL complex coacervate droplets. (a–c, e–g, i–k) Example FLIM images of the complex coacervate droplets for different ssDNA and pLL lengths. The size scale bar corresponds to 30 μm. The colour scale in (p) translates the colours in the images to fluorescence lifetimes. (d, h, l, m, n and o) Probability distribution of the sulfo-BODIPY fluorescence lifetime obtained by combining the fluorescence lifetimes from the FLIM images at 3 different positions in the sample. The pLL length is 11 (a–d), 55 (e–h) or 234 (i–l). The ssDNA length is 22 (a, e, i and m), 44 (b, f, j and n) or 88 (c, g, k and o).

Increasing the DNA chain length results in an increase of fluorescence lifetime of the sulfo-BODIPY molecular rotor (Fig. 3), especially for the longest ssDNA length (nt88). This increase in lifetime indicates that the intramolecular rotation of the molecular rotor becomes more hindered and that the local complex coacervate density thus increases for longer ssDNA lengths. This shows that the deviations from the expected *D* ∝ *N*^−1^ dependence might indeed (partly) be explained by changes in the complex coacervate density.

In contrast, changes of the pLL chain length do not affect the lifetime of the rotor (Fig. 3m, n and o), even though the changes in the pLL lengths are larger than the changes in ssDNA length. This is unexpected because in theoretical descriptions of complex coacervation,^39,40^ longer chain lengths increase both the complex coacervate density and the critical salt concentration of the complex coacervates. This critical salt concentration is the salt concentration at which the complex coacervates fall apart and where the system transitions from a two phase system (below the critical salt concentration), consisting of a polymer rich phase (the coacervate) and a diluted phase, to a one phase system (above the critical salt concentration). Earlier measurements have shown that the critical salt concentration of ssDNA/pLL coacervates increases both by increasing the ssDNA length or by increasing the pLL length.^42^ Still, we do not observe any change in fluorescence lifetime of the molecular rotor in coacervate complexes with increasing pLL length. This might mean that the pLL length has no effect at all on the complex coacervate density or that the effect of the pLL length is too small to be observed by using the sulfo-BODIPY molecular rotor. Unfortunately, the precise sensitivity of the molecular rotor is difficult to determine from calibrations with solutions with a known viscosity because the fluorescence lifetime also depends on the solvent polarity, but to give a first indication of this sensitivity: in homogeneous solutions, the fluorescence lifetime of molecular rotors show a powerlaw dependence on the macroscopic viscosity, with an exponent that can differ per molecular rotor type.^43^

Irrespective of whether no density changes occur with changing the pLL length or that the density changes are too small to measure, the effect of pLL chain length is different from ssDNA length effect: while we do not observe any effect for the pLL length, we can observe an increase in the molecular rotor fluorescence lifetime increase with increasing DNA lengths. Similar asymmetric length effects on the complex coacervate density have been observed earlier in rheology measurements, where changing the length of one of the polyelectrolytes left the storage and loss modulus nearly unaffected, while increasing the length of the oppositely charged polyelectrolyte increased the both the storage and loss modulus.^18^ A higher modulus reflects a larger crosslink density and thus an increase in coacervate density.

**Fig. 4:**
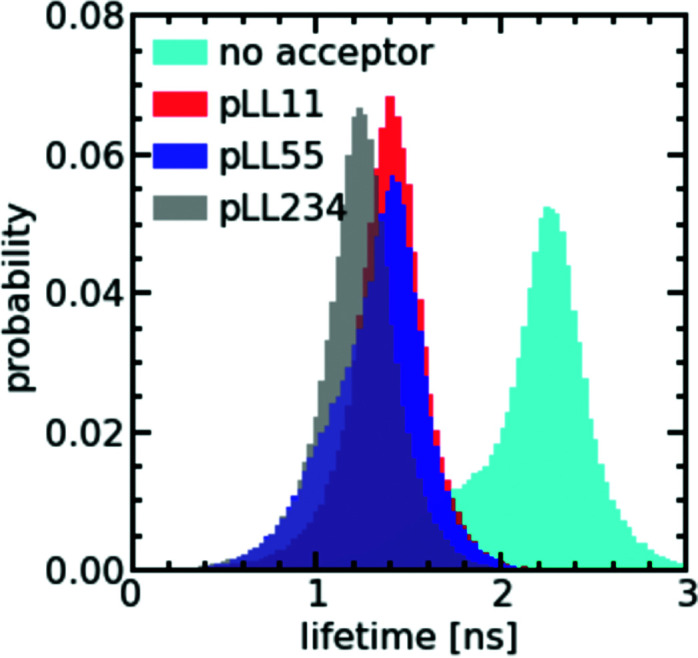
Probability distributions of the fluorescence lifetime of nt44-atto488 in ssDNA/pLL complex coacervate droplets in the presence of nt44-atto532 for different pLL lengths (pLL11, pLL55 and pLL234) and the probability distribution of the fluorescence lifetime of nt44-atto488 in ssDNA/pLL55 complex coacervate droplets in the absence of nt44-atto532 (no acceptor).

To verify whether only minor changes in complex coacervate density occur when the pLL length is changed, we have performed FRET-FLIM measurements of nt44/pLL complex coacervates containing both nt44 labelled with a donor fluorophore and nt44 labelled with an acceptor fluorophore. In FRET-FLIM measurements, the lifetime of the donor fluorophore is measured, which will become shorter when the energy transfer from the donor to the acceptor is faster. This energy transfer becomes faster when the donor and acceptor are closer to each other. An increase in complex coacervate density will decrease the average distance between the donors and acceptors and therefore will result in a decrease of the average donor fluorescence lifetime. Comparison of the fluorescence lifetime histograms for the different pLL chain lengths (Fig. 4) shows that the pLL length only has little effect on the fluorescence lifetime of the donor, suggesting that the changes in complex coacervate density are indeed small. In fact, we can use the changes in donor fluorescence lifetime to roughly estimate the density changes: the average measured fluorescence lifetime of the donor 〈*τ*_F_〉 is given by 〈*τ*_F_〉 = 1/(*k*_A_ + *k*_D_) where *k*_A_ is the energy transfer rate to the acceptor and *k*_D_ is the rate of photon emission from the donor in absence of the acceptor. The ratio between *k*_D_ and *k*_A_ depends on the distance *r* between the donor and acceptor fluorophore: *k*_D_/*k*_A_ = (*r*/*R*_F_)^6^ where *R*_F_ is the Förster radius. The ratio of transfer rate to the acceptor *k*_A_ can thus be used to calculate the changes in average distance between the donor and acceptor fluorophore: *r*_2_/*r*_1_ = (*k*_A,1_/*k*_A,2_)^1/6^. Since the coacervate density *ϕ* ∝ *r*^−3^, we get *ϕ*_2_/*ϕ*_1_ = (*k*_A,1_/*k*_A,2_)^−0.5^. With an average donor fluorescence lifetime in complex coacervates without acceptor of 〈*τ*_F_〉_donor_ = 2.1 ns (and therefore *k*_D_ = 1/〈*τ*_F_〉_donor_ ≈ 0.5 ns^−1^), an average donor fluorescence lifetime in a nt44/pLL11 complex coacervate of 〈*τ*_F_〉_pLL11_ = 1.4 ns and an average donor fluorescence lifetime in a nt44/pLL234 complex coacervate of 〈*τ*_F_〉_pLL234_ = 1.2 ns, we get *ϕ*_pLL234_/*ϕ*_pLL11_ ≈ 1.2. This factor is comparable to the density changes predicted in theoretical models for complex coacervation^39,40^ and might indicate that still minor changes in complex coacervate density occur with increasing pLL length, but that they are too small to be observed with the sulfo-BODIPY molecular rotor.

The effect of the pLL chain length on the complex coacervate density is relatively small compared to its effect on the DNA diffusion coefficient (Fig. 2): for nt44, the apparent diffusion coefficient decreased by a factor ∼3.4 when changing from pLL11 to pLL234, while the estimated density increase is only a factor ∼1.2. If changes in density are the only cause for the change in DNA diffusion coefficient with increasing pLL length, this would mean that the dynamics has to depend strongly on the complex coacervate density. In the classical model for the dynamics of associating polymers,^21^ there are two scenarios were the dynamics depends strongly on the concentration. The first scenario occurs in the sticky Rouse regime and corresponds to a transition from intramolecular to intermolecular bonds with increasing concentration. The second scenario occurs in the reptation regime, where an increase in concentration decreases the reptation tube diameter. Both scenarios do not apply to the ssDNA/pLL complex coacervates studied here. First, the reptation regime is only relevant when there are significant entanglements, which only occurs for long polyelectrolyte lengths.^41^ Here, we also see a strong length dependence for the short pLL and DNA lengths, which are too short for the reptation regime. In addition, the ionic bonds in (homo)polyelectrolyte complexes are always intermolecular since they are based on the attraction between oppositely charged species and therefore no intramolecular to intermolecular bond transition will occur. The probability of ionic bond formation can also increase with increasing concentration, but this probability *p* scales only weakly with the density (*p* ∝ *ϕ* for a theta solvent and *p* ∝ *ϕ*^1.59^ for a good solvent)^21^ and is therefore not enough to explain the relatively large change in DNA diffusion coefficient for small concentration changes. Our results thus seem to suggest that pLL length also affects the dynamics in a different way than by only changing the density. These other effects have not been discussed earlier for strongly charged polyelectrolyte complexes, but in a scaling model for weakly charged complexes a more complex length dependence has already been predicted for certain cases.^44^ Yet, we can also not exclude that the observed large difference between the density change and diffusion coefficient change is caused by experimental uncertainty. For example, there is a quite large variation in the donor fluorescence lifetime measured in the same complex coacervate (Fig. 4) and therefore the real density variation might be different from the estimated factor of 1.2. Therefore, it would be interesting to see in future research whether these small changes in density and relatively large changes in dynamics can also observed with other techniques, for example by using small angle X-ray scattering (SAXS) or small-angle neutron scattering (SANS) experiments to measure the correlation length^45^ or mesh size^46^ of the complex coacervate or rheology measurements to probe both the complex coacervate dynamics and its concentration.^18,19^

In conclusion, the chain length can have a complex effect on the density and dynamics of ssDNA/pLL complex coacervates. Changing the ssDNA chain length has a stronger effect on the coacervate density than changing the pLL length. The changes in complex coacervate density might (partly) explain the effect of the chain length on the dynamics in the complex coacervates. Yet, we cannot exclude that also other factors play a role, since the estimated changes in complex coacervate density seem relatively small compared to the observed changes in ssDNA diffusion coefficient.

### 3.3 C3Ms: monodisperse DNA exchange

The ssDNA dynamics is not only important for complex coacervate droplets, but also for complex coacervate core micelles: the ssDNA molecular exchange rate can affect the encapsulating efficiency of the C3Ms, where a slower exchange might correspond to better protected DNA and hence a better encapsulator. Therefore, we have also measured the molecular exchange of DNA between C3Ms. For this, we have used Förster resonance energy transfer (FRET). We have mixed C3Ms with part of the ssDNA fluorescently labelled with donor fluorophores and C3Ms with part of the ssDNA fluorescently labelled with acceptor fluorophores. At the start of the mixing experiment, the donors and acceptor are part of different micelle cores and are too far away from each other for FRET to occur. However, when the micelles start to exchange, the donors and acceptors can become part of the same micelle core and are close enough to each other for FRET to occur. Therefore, exchange of the micelles will result in an increase in FRET efficiency *E* in time, until the final FRET efficiency *E*(∞) is reached where the donor and acceptor micelle components are completely mixed.

**Fig. 5:**
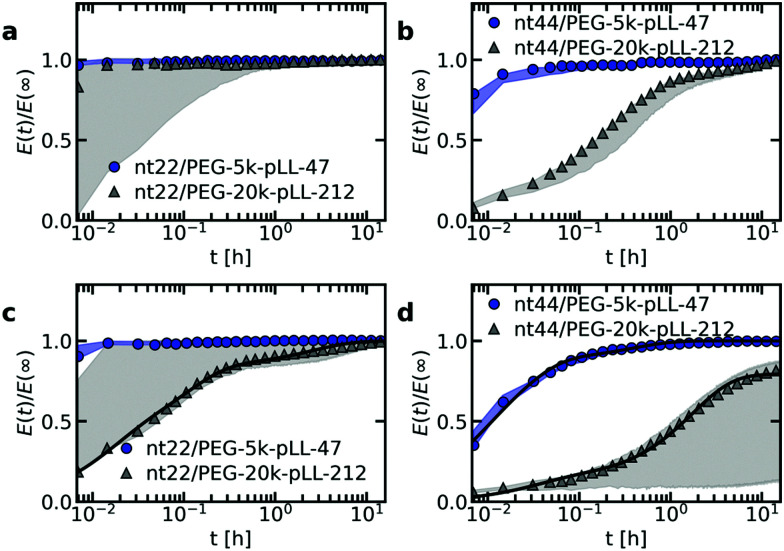
FRET-based exchange experiments of C3Ms with fluorescently labelled monodisperse DNA for different diblock copolymer lengths. In all cases, the donor fluorophore is atto488 and the acceptor fluorophore is atto532. The ssDNA length is 22 (a and c) or 44 (b and d) and the micelles are dissolved in a 1× PBS solution (a and b) or a 0.2× PBS solution (c and d). Shaded regions are an indication of the uncertainty in the exchange measurements with the region borders given by the minimum and maximum *E*(*t*)/*E*(∞) of three repetitions of the same exchange experiment. Symbols indicate an example of one of these three repetitions and solid black lines in (c) and (d) indicate fits of the data of this example exchange experiment to the analytical FRET model. The corresponding input parameters and the fit results are given in Table 1.

Both the ssDNA length and the PEG–pLL diblock copolymer length seem to affect the molecular exchange rate of DNA between C3Ms (Fig. 5a and b): for shorter chain lengths, the observed increase in FRET efficiency is faster, which indicates a faster molecular exchange rate. To better observe the differences in exchange rate for the fast exchanging C3Ms, we have also performed the exchange measurements in a 0.2× PBS solution (Fig. 5c and d) instead of a 1× PBS solution. The lower ionic strength in the 0.2× PBS solution increases the strength of the electrostatic interactions in the C3M core and therefore decreases the exchange rate.

We observe similar trends for the chain length effects on the exchange rate in both 1× PBS and 0.2× PBS solution, but we also observe very large variations between repetitions of the same experiment, as indicated by the shaded regions in Fig. 5. Because of this large uncertainty, we can conclude only that most likely both the ssDNA length and the PEG–pLL diblock copolymer length have a substantial effect on the DNA exchange rate, but we cannot exactly quantify this effect. The large variations between repetitions were unexpected: the formation of these ssDNA/PEG–pLL C3Ms is well-studied and the use of the thermal annealing protocol makes the formation of unlabelled ssDNA/PEG–pLL micelles well reproducible.^13^ Still, we expect that the origin of these large variations lies in differences in micelle preparation since the variations are much smaller for repetitions of the exchange experiments from the same micelle stock solutions (Fig. S5–S7, ESI†). These differences between micelle preparations might point to non-equilibrium micelle formation. The aim of the thermal annealing protocol was to prevent this non-equilibrium micelle formation, but maybe this protocol works insufficiently in the presence of dyes. Possibly, alternative annealing protocols, like salt annealing, could decrease the variation between the micelle preparations. Another explanation for the large variations between experiments is that differences in dye concentrations result in different exchange rates: when we compared the repetitions of the same mix experiment, we observed differences in dye concentration, in particular in the acceptor concentration (Fig. S5–S7, ESI†), which might be caused by concentration inhomogeneities in the fluorescently labelled DNA stock solutions. We will come back to this possible relation between uncertainty in the molecular exchange rate and dye concentration in the next section.

In some cases, the effect of the diblock copolymer length on the DNA exchange rate seems much larger than the effect of the polycation length on the DNA diffusion in complex coacervates: for ssDNA with a length of 44 nucleotides the measured exchange rates differ two orders of magnitude or more between PEG-5k-pLL47 and PEG-20k-pLL212 (Fig. 5b and d), while the average nt44 diffusion coefficient for pLL55 and pLL234 only differs a factor ∼1.4 (Fig. 2). This could suggest that the DNA exchange rate is not governed only by relaxation processes in the core since in that case the poly(l-lysine) length should have a similar effect on the DNA exchange rate and the DNA diffusion. Yet, for the ssDNA with a length of 22 nucleotides, the measured diblock copolymer effect is less pronounced and the variations of the exchange measurements for the different diblock copolymer are closer to each other. Therefore, to be entirely sure that the pLL chain length indeed has a much stronger effect on the molecular exchange rate than on the DNA diffusion, the uncertainty in the exchange rate determination first has to be decreased.

The measured DNA exchange rate is relatively fast compared to earlier measurements of the molecular exchange of C3Ms: the nt22/PEG–pLL47 micelles exchanged within 2 minutes, which is much faster than the exchange rate that we measured for C3Ms with poly(3-sulfopropyl methacrylate) (PSPMA) and poly(ethylene glycol)–poly(2-trimethylammonioethyl methacrylate) (PEG–PTMAEMA) at similar salt concentrations,^22^ where after 40 h the micelles were still not completely exchanged. The measured exchange rate of the nt22/PEG–pLL47 micelles is even faster than exchange rate of C3Ms with fluorescent proteins,^47^ while the protein exchange measurements were performed very close to the critical salt concentration at which the C3Ms disassemble and the nt22/PEG–pLL47 micelles are performed far away from the critical salt concentration (their critical salt concentration is ∼0.6 M NaCl^48^ and 1× PBS contains 0.137 M NaCl). In fact, the critical salt concentration is more comparable to the PSPMA/PEG–PTMAEMA micelles (∼0.79 M KCl), while the exchange rate is much faster than for the PSPMA/PEG–PTMAEMA micelles. Also for differences between the ssDNA/PEG–pLL micelles themselves, the exchange rate does not correlate with the critical salt concentration: an increase in diblock length seems to decrease the molecular exchange rates (Fig. 5), while it seems to leave the critical salt concentration unaffected.^48^ Together these observations show that the critical salt concentration is not a good predictor for the molecular exchange rate of the C3Ms.

Not only the exchange rates of the ssDNA/PEG–pLL micelles are different from the PSPMA/PEG–PTMAEMA micelles, but also the shape of exchange curve: the PSPMA/PEG–PTMAEMA showed a slow, gradual increase of the FRET efficiency as function of time plotted on logarithmic axes, while for the DNA exchange the increase is much steeper. A gradual increase on a double logarithmic scale corresponds to a broad distribution of exchange times. For the PSPMA/PEG–PTMAEMA measurements, we hypothesised that the broad range of exchange times was the result of chain polydispersity. The steeper increase for the monodisperse ssDNA exchange could support this hypothesis. Yet, we still observe different time scales for the C3Ms with slow monodisperse ssDNA exchange: there is an initial increase that is too fast to be measured and afterwards a second exchange time scale seems to occur. Subsequently, we observe a quasi-plateau and then the FRET efficiency increases again at longer logarithmic time scales. The initial fast exchange might correspond to a small population of unstable micelles, for example because these micelles are destabilised by the shear of mixing of donor and acceptor micelle solutions. A possible explanation for the two other time scales is that the ssDNA labelled with donor fluorophores have a different exchange rate than the ssDNA labelled with acceptor fluorophores.

As a first check of this explanation for the different time scales in the monodisperse ssDNA exchange, we have fitted the C3M exchange experiments with an analytical model for FRET-based micelle exchange experiments, which we developed recently.^22^ In this case, both the donor and acceptor fluorophores consisted of two different species, where the exchange rate of the fast exchanging species was the same for the donor and acceptor, while the exchange rate of the slow exchanging species could be different for the donor and acceptor. Further details of this fit procedure given in the materials and methods (Section 2.10). We note that the purpose of this fit is to explain the shape of the DNA exchange experiments curves and not to exactly determine the rate constants because the experimental uncertainty is too large for this. The precise values of the rate constants obtained from the fit (Table 1) are therefore not that relevant. The fits can describe the exchange experiments well (Fig. 5c and d), which suggests that the ssDNA exchange indeed occurs with only three different exchange rates instead of a broad distribution.

**Table 1:** Fit input parameters (*N*, *α* and *ν*) and fit results (*x*_fast_, *k*_D_ and *k*_A_) of fluorescently labelled ssDNA exchange for C3Ms in a 0.2× PBS solution with atto488 as donor fluorophore label and atto532 as acceptor fluorophore label. The exchange of nt22/PEG-5k-pLL47 was too fast to fit the data and therefore no fit results are given for this sample

Sample	*N*	*α*	*ν*	*x*_fast_	*k*_fast_ (h^−1^)	*k*_D_ (h^−1^)	*k*_A_ (h^−1^)
nt22/PEG-5k-pLL47	2454	0.03	0.01	—	—	—	—
nt22/PEG-20k-pLL212	3835	0.03	0.01	0.2	62	9	0.2
nt44/PEG-5k-pLL47	1079	0.06	0.01	0.2	133	41	1
nt44/PEG-20k-pLL212	1702	0.06	0.01	0.07	30	0.6	0.001

### 3.4 Dye effect on the molecular exchange of C3Ms

**Fig. 6:**
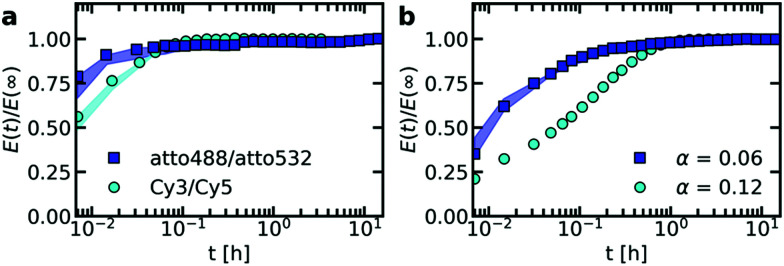
Effect of the fluorescent dye on the exchange rate of nt44/PEG-5k-pLL47 C3Ms with fluorescently labelled DNA. (a) Effect of the dye type: FRET-based exchange experiments of nt44/PEG-5k-pLL47 C3Ms in 1× PBS solution with an atto488/atto532 FRET-pair or an cyanine3/cyanine5 (Cy3/Cy5) FRET-pair. In both cases, the fraction of labelled chains *α* is 0.06 (b) effect of the dye concentration: FRET-based exchange experiments of nt44/PEG-5k-pLL47 C3Ms in 0.2× PBS solution with an atto488/atto532 FRET-pair and a fraction of labelled chains *α* of *α* = 0.06 or *α* = 0.12. Shaded regions in both (a) and (b) are an indication of the uncertainty in the exchange measurements with the region borders given by the minimum and maximum *E*(*t*)/*E*(∞) of three (for atto488/atto532, *α* = 0.06, 1× PBS and 0.2× PBS) or two (for Cy3/Cy5) repetitions of the same exchange experiment. Symbols indicate an example of one of these repetitions. The exchange experiment with a label fraction *α* = 0.12 in (b) was performed only once and therefore the uncertainty is not indicated.

To verify that the fluorophore label can indeed affect the exchange rate of the ssDNA between C3Ms, we have performed the exchange experiment with a cyanine3/cyanine5 (Cy3/Cy5) FRET pair instead of the atto488/atto532 FRET pair. We indeed observe a change in the exchange rate of nt44/PEG-5k-pLL47 micelle, with a slower exchange for the Cy3/Cy5 FRET pair (Fig. 6a). Similar effects occur for the exchange of nt44/PEG-20k-pLL212 micelles (Fig. S8, ESI†). The Cy3/Cy5 pair is more hydrophobic: the partition coefficient of cyanine5 in anionic lipids is more than 10^4^ times higher than for atto532 and more than 3 × 10^4^ times higher than for atto488.^49^ This suggests that the use of a more hydrophobic dye decreases the exchange rate. This could also explain why the acceptor rate would be lower than the donor exchange rate, as suggested by the fits (Table 1), since atto532 might be slightly more hydrophobic than atto488.

To further study the dye effect, we have also increased the dye concentration by a factor 2. A larger dye concentration results in a slower exchange (Fig. 6b). This shows that variations in dye concentration can indeed affect the exchange rate, as we suggested in the previous section to explain the large variations in exchange rate between repetitions of the same measurements. However, for the repetitions of the same exchange experiments the dye concentration effect seems to be reversed and the micelles with a lower acceptor concentration seem to exchange more slowly (Fig. S5–S7, ESI†). This could point to a complex dye effect where for example the acceptor concentration has a different effect on the exchange rate than the donor concentration. Alternatively, except from the dyes, also other effects in the micelle preparation might have played a role, like the formation of non-equilibrated micelles. To test whether other factors than the dyes play a role in the large variations in exchange experiments, the ssDNA/PEG–pLL exchange could be measured also by using small angle neutron scattering (SANS) since for these measurements no dyes are needed.

The large effects of the dyes on the molecular exchange rates of C3Ms show that one has to be careful with interpreting the results of FRET-based micelle exchange experiments and that in general SANS measurements are a better option to measure the exchange: SANS measurements are based on neutron scattering contrast and the introduction of deuterated protons will have a much smaller effect on the exchange rate than the introduction of dyes. Although the dye effect is a large disadvantage for fundamental studies to the molecular exchange of C3Ms, it shows that the molecular exchange can be tuned by just introducing an additional hydrophobic group. It was already shown earlier that additional hydrophobicity can affect the static properties of both complex coacervates^50^ and complex coacervate core micelles.^51^ In addition, by simulating the C3M exchange with Langevin dynamics simulations, we showed that the non-electrostatic attraction (like hydrophobic interactions) between the oppositely charged polyelectrolytes, can largely affect the exchange rate.^31^ These simulations also suggested that the non-electrostatic interactions between only one of the core species might not be enough to reduce the exchange rate. In that respect, it would be interesting to see whether the dyes have similar effects on the molecular exchange of other C3Ms systems or that also specific interactions with the poly(l-lysine) and/or the ssDNA have played a role in the large dye effect that we observed here.

### 3.5 C3Ms: polydisperse diblock exchange

**Fig. 7:**
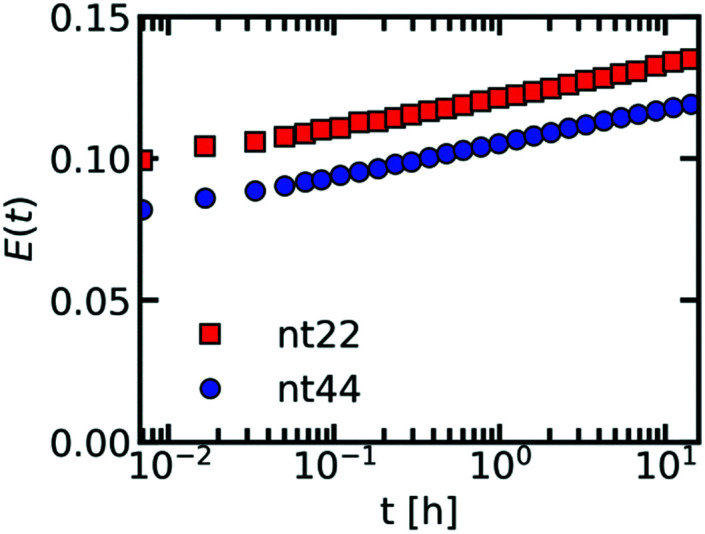
FRET-based exchange experiments of C3Ms with fluorescently labelled polydisperse PEG-5k-pLL47 diblock copolymers for two different ssDNA lengths. Sulfo-cyanine 3 is used as the donor fluorophore and sulfo-cyanine 5 is used as the acceptor fluorophore. The micelles are dissolved in a 1× PBS solution.

So far, we have focused on the molecular exchange of DNA. We have shown that the monodisperse ssDNA exchanges with only a few different rates, which we explained by the DNA being monodisperse and the different rates being caused by differences between the donor and acceptor exchange (and by a small fraction of unstable C3Ms). Apart from the ssDNA chains, the C3Ms also consists of oppositely charged PEG–pLL diblock copolymers, which are polydisperse. If the low number of exchange rates observed for ssDNA is indeed the result of the DNA being monodisperse, we should observe a broader distribution of exchange rates for the PEG–pLL diblock copolymer. To verify this, we have fluorescently labelled the PEG–pLL diblock copolymer and measured their molecular exchange (Fig. 7). In this case, we did not divide by the FRET efficiency of the completely mixed micelles because the FRET efficiency that we measured by directly preparing micelles with donor and acceptor fluorophores in their core, was lower than we measured at the end of the exchange experiments. This uncertainty in end FRET efficiency determination can be explained by the fact that the overall FRET efficiency of these fluorescently labelled PEG–pLL micelles is relatively low. We note that because we did not divide by the final FRET efficiency, the exchange experiments of the two different DNA lengths cannot be directly compared to each other. We can conclude only that the shape of the exchange curve is consistent for the different DNA lengths and that the broad distribution of exchange rates that underlie this shape is a common feature of the PEG–pLL exchange.

We indeed observe a gradual increase in FRET efficiency for the C3Ms with fluorescently labelled PEG–pLL diblock copolymers, similar to what we observed earlier for the PSPMA exchange of PSPMA/PEG–PTMAEMA micelles.^22^ This further supports that chain polydispersity is the main cause for a broad distribution of exchange rates observed within a single exchange experiment. The difference between the ssDNA and PEG–pLL exchange also shows that the exchange of one of the core species cannot be used as a direct measure for the exchange of the oppositely charged core species.

The differences between the shape in the monodisperse ssDNA and polydisperse diblock copolymer exchange curves can be explained in two ways. In the first explanation, the ssDNA and the diblock copolymer exchange independently from each other. This explanation is unlikely because this does not agree with the combined exchange that we observed earlier in Langevin dynamics simulations.^31^ In addition, the diblock copolymer length seems to have a substantial effect on the ssDNA exchange rate (Fig. 5), which we do not expect for independent exchange. In the second explanation, the ssDNA and diblock copolymer exchange together, but the exchange of neutrally charged complexes is preferred and therefore the ssDNA exchanges mainly with polydiblock copolymers with a polycationic block length that is the same as (a multiple of) the ssDNA length. These diblock copolymers therefore have the largest exchange rate. Diblock copolymers with polycationic block lengths that result in the formation of complexes with a net charge will exchange less often and therefore have a lower exchange rate. In this way, the average exchange rate of all monodisperse DNA molecules can be the same, while the exchange rates of the polydisperse PEG–pLL diblock copolymers can differ depending on their polycationic length.

### 3.6 Molecular exchange mechanism of C3Ms

**Fig. 8:**
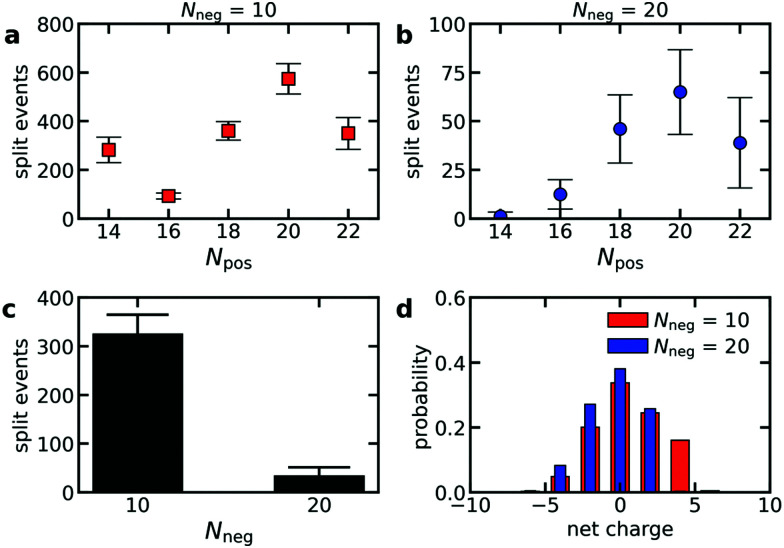
Langevin dynamics simulations of the exchange of C3Ms consisting of monodisperse anionic homopolymers and cationic–neutral diblock copolymers with a polydisperse cationic block. (a and b) Average number of split events per diblock copolymer in the time range 2 × 10^6^*τ*_s_ to 2 × 10^7^*τ*_s_ as function of the cationic block length *N*_pos_ for an anionic homopolymer length *N*_neg_ = 10 (a) or *N*_neg_ = 20 (b). Error bars indicate the standard deviation of the average. (c) The average number of split events per anionic homopolymer in the time range 2 × 10^6^*τ*_s_ to 2 × 10^7^*τ*_s_ for simulations with *N*_neg_ = 10 and simulations with *N*_neg_ = 20. Error bars again indicate the standard deviation of the average. (d) Probability distribution of the net charge of the complexes that split off in the time range 2 × 10^6^*τ*_s_ to 2 × 10^7^*τ*_s_.

To verify that polydispersity of the diblock copolymers can result in different diblock copolymer exchange rates even when the oppositely charged polyelectrolyte is monodisperse, we have performed Langevin dynamics of C3Ms consisting of monodisperse polyanions and diblock copolymers with different polycation lengths. Indeed, the exchange rate differs for the different cationic block lengths *N*_pos_, with the highest exchange rates for the diblock copolymers that can form neutrally charged complexes with the oppositely charged homopolymer (Fig. 8a and b). This is also reflected by the probability distribution of the net charge of the complexes that split off during the simulations, with the highest probability for neutrally charged complexes (Fig. 8d). The charge probability distribution also shows that all exchange events involve at least one polycation and polyanion and that no independent exchange of the polycations or polyanions occurs. Besides the charge ratio between the polycationic block and the polyanion(s), the absolute polyanion length also has a large effect on the exchange rate: an increase of the polyanion length *N*_neg_ from 10 to 20 results in a much lower exchange rate (Fig. 8a–c). This agrees with the observed decrease in exchange rate when doubling the ssDNA length in ssDNA exchange experiments (Fig. 5).

The fact that the charge ratio between the cationic block and the polyanion(s) has a larger impact on the diblock copolymer exchange rate than the cationic block length itself (Fig. 8a and b) indicates that the exchange rate is not only governed by the polyelectrolyte movements in the C3M core: inside a complex coacervate, the oppositely charged polyelectrolytes do not move simultaneously, as shown by different diffusion coefficients of oppositely charged polyelectrolytes in the same complex,^52^ and therefore for movements inside the C3M core, the absolute length of the polyelectrolytes should be more important than the charge ratio. Instead, the large dependence on the charge ratio suggests that also an activated process is involved in the exchange where the height of the energy barrier is mainly determined by the number of uncompensated charges in the expelled complex.

In contrast, the strong dependence of the exchange rate on the polyanion length, shows that the exchange rate is also not only governed by an activated process. For example, the same number of monomers is involved in the exchange of a polycationic block with *N*_pos_ = 20 with the oppositely charged polyanion(s) with length *N*_neg_ = 10 or *N*_neg_ = 20. Therefore, the energy barrier is the same for both polyanion lengths. Still, we observe a much larger exchange rate for the shorter polyanion (Fig. 8a and b). Similar effects occur for other cationic block lengths and we have also observed the same effects in exchange experiments with fluorescently labelled ssDNA (Fig. 5). This indicates that relaxation processes in the C3M core also affect the exchange rate.

The chain length effect on the core relaxation part of the C3M exchange cannot be described by a simple (sticky) Rouse relaxation: for the polyanion, the average number of split events with a cationic block with length *N*_pos_ = 20 decreases by a factor ∼8.8 when increasing the polyanion length from *N*_neg_ = 10 to *N*_neg_ = 20, which is larger than the *τ* ∝ *N*^2^ dependence of the sticky Rouse model. In contrast, increasing the cationic block length from *N*_pos_ = 18 to *N*_pos_ = 22, decreases the number of split events only by a factor of ∼1.03 (*N*_neg_ = 10) or ∼1.3 (*N*_neg_ = 20), while in the sticky Rouse model this should be a factor 1.5 and this factor can even become larger when also the effect of non-electrostatic interactions on the energy barrier is taken into account. At the moment, we cannot exactly explain this complex effect of the chain length on the core relaxation part of the exchange. Possibly, the average number of chains to which a polyelectrolyte binds partly determines the exchange rate. Shorter chains have a smaller radius of gyration and therefore bind to less chains on average, which might increase the expulsion rate. In that case, density effects might have further enhanced the effect of the polyanion length since a lower density could further decrease the average number of chains to which a polyelectrolyte binds. This density effect did not occur in the comparison of the cationic block length effect because here we compared cationic block lengths that are part of the same C3Ms and therefore the density is the same for both polycation lengths.

**Fig. 9:**
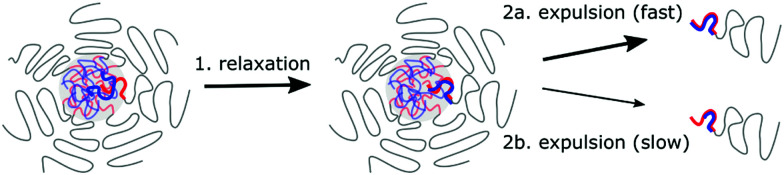
Schematic overview of the C3M exchange mechanism: (1) Relaxation processes in the C3M core are required to form a separate small polyelectrolyte complex inside the core. (2) This separate small complex can subsequently be expelled from the core. This is an activated process and the expulsion probability is large when the complex is neutrally charged (2a) and small when the complex has a large net charge (2b).

In summary, both an activated process and relaxation processes in the core determine the C3M exchange rate (Fig. 9), as was also suggested for the exchange of amphiphilic diblock copolymers micelles.^53^ However, the underlying mechanism of the C3M exchange are different than for the amphiphilic diblock copolymer micelles: for the C3M exchange, the exchange rate of a core species depends also on the length of the oppositely charged core species, while the exchange of an amphiphilic diblock copolymer only depends on its own length because there is only one core species. As a result, the energy barrier of the activated process in C3M exchange might not always increase with increasing polyelectrolyte length and the charge ratio can be more important than the absolute polyelectrolyte length. In addition, the contribution of the relaxation in the core to the exchange rate deviates from the Rouse relaxation suggested for amphiphilic diblock copolymer micelles. These differences between C3Ms and amphiphilic diblock copolymer micelles can explain why we could not use the amphiphilic diblock copolymer model to describe the exchange of PSPMA/PEG–PTMAEMA micelles^22^ and why in a recent SANS study of C3M exchange a lower polydispersity was needed than measured to obtain a good fit between the exchange data and the amphiphilic diblock copolymer model.^24^ Furthermore, these differences stress the importance of specifically studying C3Ms to further unravel the effect of chain lengths and other parameters on the C3M exchange rate.

## 4 Conclusions

In conclusion, both the length of the oppositely charged poly(l-lysine) and the DNA length itself affect the DNA dynamics in both ssDNA/pLL complex coacervate droplets and ssDNA/PEG–pLL complex coacervate core micelles. For the complex coacervate droplets, the DNA diffusion coefficient showed a stronger dependence on its chain length than predicted by the sticky Rouse model. We have shown that this stronger dependence can be (partly) explained by an increase in complex coacervate density with increasing DNA length, but also other factors might have played a role.

For complex coacervate core micelles, the chain length effects were more difficult to quantify because we observed large variations in C3M exchange rates between repetitions of the same experiment. We hypothesise that these large variations are caused by dye concentration variations and we have shown that changing the dye concentration can indeed affect the C3M exchange rate. In addition, we have shown that the dye type can also affect the exchange rate and we have hypothesised that the different exchange rates observed for the exchange of monodisperse DNA are mainly caused by a differences in exchange rate for DNA labelled with a donor fluorophore and DNA labelled with an acceptor fluorophore. Measuring the exchange of ssDNA/PEG–pLL micelles with small angle neutron scattering (SANS) could be used to test our hypotheses on the dye effects.

Although the determination of the exact values of the C3M exchange rate constants was hindered by the large uncertainty of the measurements, the shape of the FRET-based C3M exchange curve still provided information on the exchange mechanisms: the broader distribution of exchange rates for the polydisperse PEG–pLL diblock copolymer than the monodisperse ssDNA suggests that chain length polydispersity is the main cause for a broad distribution of exchange times. The different exchange rates for ssDNA and PEG–pLL can be explained by a preferred exchange of ssDNA with PEG–pLL with a cationic block length that is the same as (a multiple of) the ssDNA length, which results in a lower exchange rate for PEG–pLL polymers with mismatched cationic block lengths. We have supported this explanation by performing Langevin dynamics simulations and we have shown that both relaxation processes in the core and an activated process determine the C3M exchange rate. Furthermore, we have concluded that the chain length effect on the C3M exchange is different from the chain length effect on the exchange of amphiphilic diblock copolymers. For C3Ms, the expulsion of neutral complexes is preferred and as a result the length ratio of the oppositely charged core species might play a larger role than the absolute chain length. Therefore, the length ratio of the oppositely charged polyelectrolytes might be used to tune exchange rate. However, the presence of polydisperse polymers make it more difficult to use this length ratio as tuning parameter since a mismatch in only the average chain length is not enough to prevent fast exchange, as was illustrated here by the fast ssDNA exchange in the presence of polydisperse PEG–pLL. Apart from the length ratio with the oppositely charged cationic block, the ssDNA length itself can also be used to adapt the exchange rate, with a lower exchange rate for longer ssDNA lengths.

## Conflicts of interest

There are no conflicts to declare.

## Supplementary Material

SM-018-D1SM01787J-s001
