# Supplementary material for: DNA dynamics in complex coacervate droplets and micelles
Source: Soft Matter. 2022 Feb 17;18(10):2012–27. doi: 10.1039/d1sm01787j (PMC8905490; doi:10.1039/d1sm01787j)
Supplement: SM-018-D1SM01787J-s001 [file SM-018-D1SM01787J-s001.pdf]

Electronic Supplementary Material (ESI) for Soft Matter.
This journal is © The Royal Society of Chemistry 2022

Supporting Information:
DNA dynamics in complex coacervate droplets and micelles

Inge Bos, Eline Brink, Lucile Michels, and Joris Sprakel *

Physical Chemistry and Soft Matter, Wageningen University & Research, Stippeneng 4, 6708 WE, Wageningen, The Netherlands

S1 Oligonucleotide sequences

Table S1. The oligonucleotide sequences used in this study.

Name	Sequence (5'-3')
nt22	TCAACATCAGTCTGATAAGCTA
nt44	TCAACATCAGTCTGATAAGCTATGGATACTCGTCTGGACTACTT
nt88	TCAACATCAGTCTGATAAGCTATGGATACTCGTCTGGACTACTT<br>ACTCACTCATTCATCACTATCTACCGTCGCATTCAGCATTCATG

S2 FRAP bleach profile

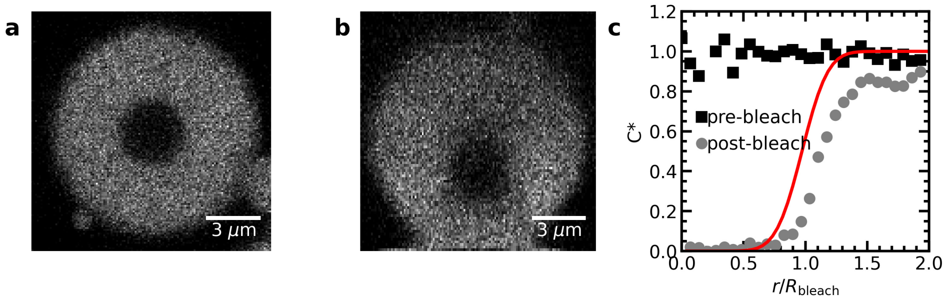

Figure S1. Profile of the bleach spot in a FRAP measurement of a nt88/pLL234 complex coacervate sample. a) xy-image of the droplet with the bleach spot b) xz-image of the droplet and bleach spot. c) Comparison of the radially averaged intensity in the xy-plane before and directly after bleaching as function of distance r from the center of the bleach spot. The red solid line shows the best fit of the post-bleach profile to the radial profile of the 3D infinite model.[1] The fit parameter is the normalised time $t^* = t/\tau$. This t^* is 0 for a perfect step bleach profile without any diffusion during the bleaching. In this case, $t^* = 0.01$.

*Corresponding author: joris.sprakel@wur.nl

S3 Deconvolution of the emission spectra

To determine the relative contributions of the donor emission and acceptor emission to the total emission spectrum, we have used the same approach as previously[2] and we have fit the total spectrum with a weighted sum of the separate donor and acceptor spectra:

$$I_{\mathrm{tot}}(\lambda) = dI_D(\lambda) + aI_A(\lambda) \tag{S1}$$

Here $I_{\mathrm{tot}}(\lambda)$ it the total intensity at a wavelength λ and $I_D(\lambda)$ and $I_A(\lambda)$ are the intensity of the donor and acceptor spectrum at this wavelength respectively. The prefactors d and a give the relative contribution of the donor and acceptor. These prefactors are the fit parameters. To perform this fit, we have first approximated both the donor and acceptor emission spectrum with a sum of a two log normal functions:

$$I(\lambda) = \frac{x}{\lambda\sigma_1\sqrt{2\pi}} \exp\left(-\frac{(\ln(\lambda)-\mu_1)^2}{2\sigma_1^2}\right) + \frac{1-x}{\lambda\sigma_2\sqrt{2\pi}} \exp\left(-\frac{(\ln(\lambda)-\mu_2)^2}{2\sigma_2^2}\right) \tag{S2}$$

here $I(\lambda)$ is the intensity of the donor or acceptor. Every dye has its own specific values for x, σ_1, μ_1, σ_2 and μ_2, which are given in Table S2. These values were obtained by fitting Equation S2 to the donor or acceptor emission spectrum using a Monte Carlo fit algorithm.

Table S2. Overview of the parameters used to describe the dye emission spectrum with Equation S2.

dye	x	μ_1	σ_1	μ_2	σ_2
atto488	0.58	6.26	0.026	6.31	0.046
atto532	0.50	6.32	0.021	6.37	0.050
cyanine3	0.53	6.34	0.019	6.39	0.040
cyanine5	0.68	6.51	0.022	6.55	0.053
sulfo-cyanine3	0.54	6.35	0.020	6.41	0.039
sulfo-cyanine5	0.36	6.51	0.019	6.52	0.042

To fit Equation S1 to the measured emission spectra, we have used the non-linear least square fit algorithm as implemented in the python package SciPy. An example fit for every FRET pair is given in Fig. S2. After fitting the data, the total donor intensity and total acceptor intensity, needed to calculate the FRET efficiency, were obtained by integrating $dI_D(\lambda)$ and $aI_A(\lambda)$ over the wavelength range $\lambda = 400\,\mathrm{nm}$ to $\lambda = 700\,\mathrm{nm}$ (atto488/atto532 dyes) or $\lambda = 500\,\mathrm{nm}$ to $\lambda = 800\,\mathrm{nm}$ (cyanine3/cynanine5 and sulfo-cyanine3/sulfo-cyanine5 dyes).

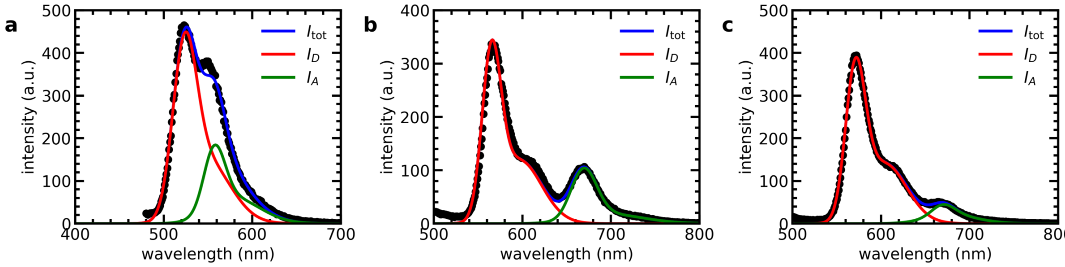

Figure S2. Example of fluorescence emission spectra of nt44/PEG-5k-pLL47 micelles with (a) part of the nt44 labelled with atto488 dye and a part of the nt44 labelled with atto532 dye, (b) part of the nt44 labelled with cyanine 3 dye and a part of the nt44 labelled with cyanine 5 dye and (c) part of the PEG-5k-pLL47 labelled with sulfo-cyanine 3 dye and a part of the PEG-5k-pLL47 labelled with sulfo-cyanine 5 dye. Solid lines indicate fits of the spectra to Equation S1 where $I_D(\lambda)$ and $I_A(\lambda)$ are defined by Equation S2 with the parameters given in Table S2.

S4 Light scattering measurements

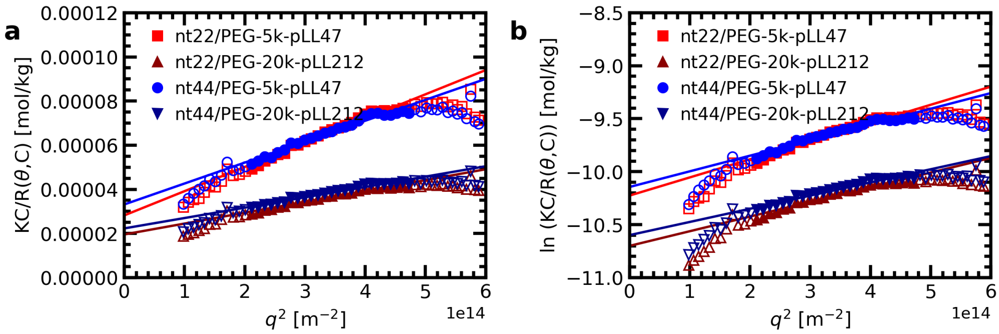

Figure S3. (a) Zimm plot and (b) Guinier plot for light scattering data of ssDNA/pegpLL micelles in a 0.2X PBS solution. The solid lines indicate the linear fit of the data from which the intercept and thus the micelle molar mass was estimated. For this fit, only the data points marked with solid symbols are used. The micelle aggregation numbers N calculated from the molar mass obtained from these fits are given in Table 1 of the main text. For this, the average of the molar mass obtained from the Zimm analysis and the molar mass obtained from the Guinier analysis is used.

S5 Estimation of the geometric constant ν

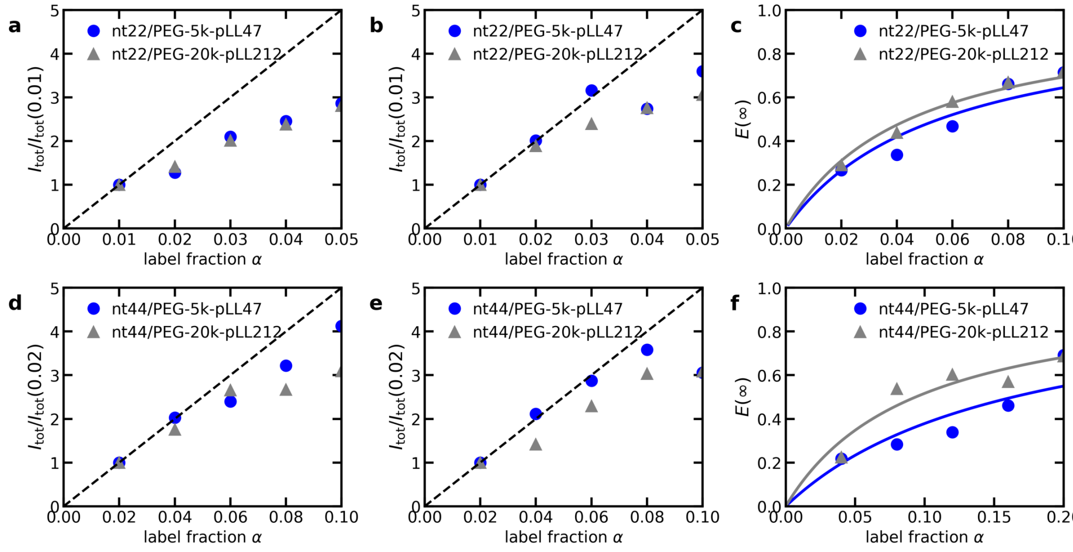

Figure S4. Fluorescence of the equilibrated ssDNA/pegpLL micelles at different label fractions in a 0.2X PBS solution. (a,b,d,e) Fluorescence intensity of the donor micelles (a,d) and acceptor micelles (b,e) with nt22 (a,b) or nt44 (d,e) at different label fractions α normalised to the intensity at a label fraction $\alpha = 0.01$ (a,b) or $\alpha = 0.02$ (d,e). The dashed lines indicate the theoretical intensity without self-quenching. (c,f) FRET efficiency of the mixed micelles with nt22 (c) or nt44 (f) as a function of label fraction. FRET efficiencies are corrected for differences in donor and acceptor self-quenching. Solid lines indicate fits with Equation 8 of the main text to obtain the geometric constant ν. The used values for the micellel aggregation number N (needed to calculate $n_A = 0.5N\alpha$) and the obtained values for the geometric constant ν are indicated in Table 1 of the main text.

S6 Comparison of repetitions of C3Ms exchange experiments

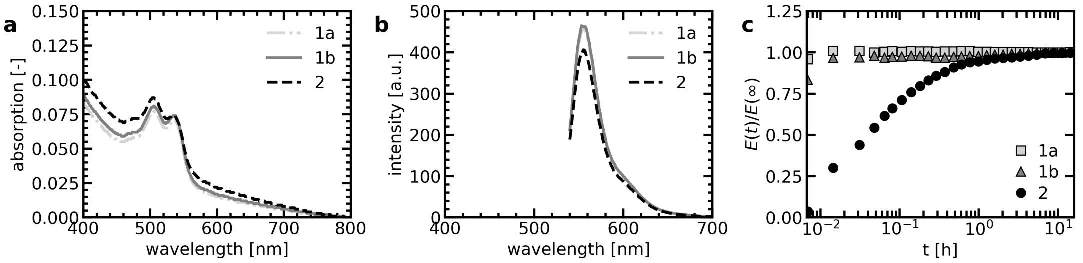

Figure S5. Comparison of three repetitions of the mix experiment of nt22/PEG-20k-pLL212 micelles in 1X PBS with part of the ssDNA fluorescently labelled with atto488 and atto532. For the 1a and 1b mix experiments the same micelle stock solutions are used, while for mix experiment 2 other micelle stock solutions are used than in 1a and 1b. (a) Absorption spectra of the micelles after the mix experiments. The background absorbance that increases with decreasing wavelength is probably the result of light scattering by the micelles. (b) Acceptor emission spectra after the mix experiments (excitation wavelength: 530 nm). (c) The mix experiments: the FRET efficiency E at time t divided by the FRET efficiency of completely mixed micelles $E(\infty)$.

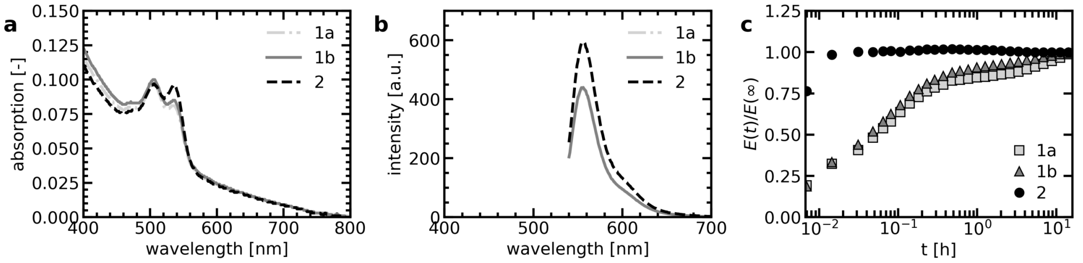

Figure S6. Comparison of three repetitions of the mix experiment of nt22/PEG-20k-pLL212 micelles in 0.2X PBS with part of the ssDNA fluorescently labelled with atto488 and atto532. For the 1a and 1b mix experiments the same micelle stock solutions are used, while for mix experiment 2 other micelle stock solutions are used than in 1a and 1b. (a) Absorption spectra of the micelles after the mix experiments. The background absorbance that increases with decreasing wavelength is probably the result of light scattering by the micelles. (b) Acceptor emission spectra after the mix experiments (excitation wavelength: 530 nm). (c) The mix experiments: the FRET efficiency E at time t divided by the FRET efficiency of completely mixed micelles $E(\infty)$.

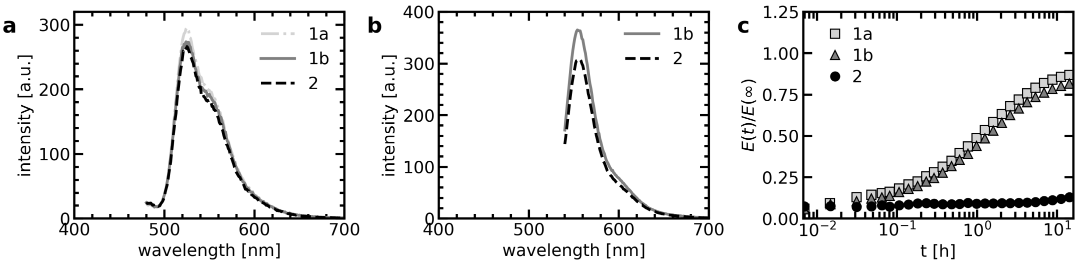

Figure S7. Comparison of three repetitions of the mix experiment of nt44/PEG-20k-pLL212 micelles in 0.2X PBS with part of the ssDNA fluorescently labelled with atto488 and atto532. For the 1a and 1b mix experiments the same micelle stock solutions are used, while for mix experiment 2 other micelle stock solutions are used than in 1a and 1b. (a) Emission spectra at the start of the mixing experiment ($t \approx 25\,\mathrm{s}$). (b) Acceptor emission spectra after the mix experiments (excitation wavelength: 530 nm). (c) The mix experiments: the FRET efficiency E at time t divided by the FRET efficiency of completely mixed micelles $E(\infty)$ where $E(\infty)$ was obtained from a separate measurement of the FRET efficiency of nt44/PEG-20k-pLL212 C3Ms that were prepared to have the donor and acceptor fluorophores completely mixed in their core.

S7 Dye effect on the exchange of nt44/PEG-20k-pLL212 micelles

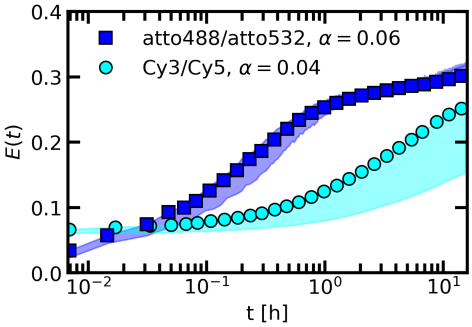

Figure S8. Effect of the fluorescent dye type on the exchange rate of nt44/PEG-20k-pLL212 C3Ms with fluorescently labelled DNA: comparison of the FRET efficiency E as function of time after mixing t for an atto488/atto532 FRET-pair (label fraction $\alpha = 0.06$) and a cyanine3/cyanine5 (Cy3/Cy5) FRET-pair (label fraction $\alpha = 0.04$). In both cases, the micelles are dissolved in 1X PBS solution. Shaded regions are an indication of the uncertainty in the exchange measurements with the region borders given by the minimum and maximum $E(t)/E(\infty)$ of three (for atto488/atto532) or two (for Cy3/Cy5) repetitions of the same exchange experiment. Symbols indicate an example of one of these repetitions.

References

(1) Taylor, N. O.; Wei, M.-T.; Stone, H. A.; Brangwynne, C. P. Quantifying dynamics in phase-separated condensates using fluorescence recovery after photobleaching. *Biophys. J.* **2019**, *117*, 1285–1300.

(2) Bos, I.; Timmerman, M.; Sprakel, J. FRET-based determination of the exchange dynamics of complex coacervate core micelles. *Macromolecules* **2021**, *54*, 398–411.
